# Psychotropic Drugs for the Management of Chronic Pain and Itch

**DOI:** 10.3390/ph12020099

**Published:** 2019-06-24

**Authors:** Daria A. Belinskaia, Mariia A. Belinskaia, Oleg I. Barygin, Nina P. Vanchakova, Natalia N. Shestakova

**Affiliations:** 1 Sechenov Institute of Evolutionary Physiology and Biochemistry, Russian Academy of Sciences, pr. Torez 44, St. Petersburg 194223, Russia; oleg_barygin@mail.ru (O.I.B.); n_shestakova@list.ru (N.N.S.); 2 International Centre for Neurotherapeutics, Dublin City University, Glasnevin, Dublin 9, Ireland; belinskaya88@gmail.com; 3 Department of Pedagogy and Psychology, Faculty of Postgraduate Education, First Pavlov State Medical University, L’va Tolstogo str. 6-8, St. Petersburg 197022, Russia; vanchakova@spb-gmu.ru

**Keywords:** chronic pain, psychotropic drugs, pharmacological profile, animal model, clinical trial

## Abstract

Clinical observations have shown that patients with chronic neuropathic pain or itch exhibit symptoms of increased anxiety, depression and cognitive impairment. Such patients need corrective therapy with antidepressants, antipsychotics or anticonvulsants. It is known that some psychotropic drugs are also effective for the treatment of neuropathic pain and pruritus syndromes due to interaction with the secondary molecular targets. Our own clinical studies have identified antipruritic and/or analgesic efficacy of the following compounds: tianeptine (atypical tricyclic antidepressant), citalopram (selective serotonin reuptake inhibitor), mianserin (tetracyclic antidepressant), carbamazepine (anticonvulsant), trazodone (serotonin antagonist and reuptake inhibitor), and chlorprothixene (antipsychotic). Venlafaxine (serotonin-norepinephrine reuptake inhibitor) is known to have an analgesic effect too. The mechanism of such effect of these drugs is not fully understood. Herein we review and correlate the literature data on analgesic/antipruritic activity with pharmacological profile of these compounds.

## 1. Overview

Chronic pain accompanies many pathological conditions, such as diabetes, chronic renal failure, and cancer [1,2,3]. The nature of chronic pruritus is currently under discussion, but there are reasons to believe that it can be assumed as a specific type of pain [4,5].

At least half of patients suffering from chronic pain and itching are diagnosed with mental disorders such as depression and/or anxiety [6,7]. At the same time, chronic pain and pruritus syndromes are found in 60% of patients with depression [8,9]. Regardless of whether the mental disorders are primarily or secondarily related to chronic pain and itching, such patients need compensatory therapy with psychotropic drugs, some of which have their own analgesic and antipruritic action [10,11]. It is indicated often in medical literature that it is the class of tricyclic antidepressants (TCA) that have such efficacy. However, effective analgesic/antipruritic drugs are found among practically all antidepressant groups [12]. Anticonvulsants and antipsychotics are widely used in pain management, too [13,14].

**Figure 1:**
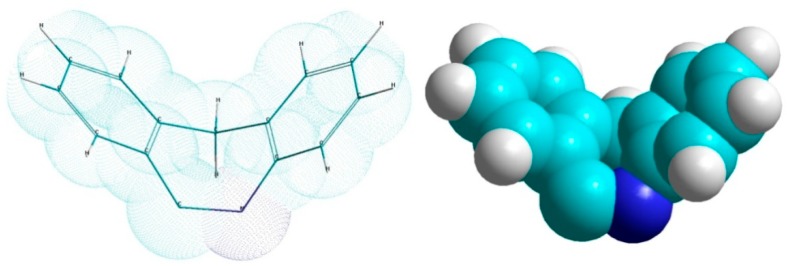
V-like conformation of aromatic rings in the example of mianserin.

Generally, the detection of analgesic and especially antipruritic effects of psychotropics is random, there are no certain structure criteria for determining their efficacy. In our previous studies, using molecular modelling methods, we showed that the tricyclic and tetracyclic psychotropic drugs that have the V-like conformation of their heterocyclic group (aromatic rings co-centered at an angle of 120–160 degrees, Figure 1) have analgesic and antipruritic activity, whereas the drugs that have a plain conformation of their rings do not [15]. However, the target of analgesic and antipruritic action of the effective compounds remained unclear.

According to the data reported in literature, some TCAs are blockers of the *N*-methyl-D-aspartate receptors (NMDAR), which are involved in the pain signal pathways [16,17]. In 2009, using the patch-clamp technique, Barygin and co-authors showed that the tricyclic compounds that have V-like configuration of their rings are able to block NMDAR through a voltage-dependent mechanism, unlike plain compounds [18]. We hypothesised that it is the anti-glutamate mechanism that determines the analgesic and antipruritic efficacy of psychotropic drugs containing the V-like group. Later, using electrophysiological methods, we showed that the antidepressants desipramine and amitriptyline as well as antipsychotics chlorpromazine and atomoxetine, which contain the V-like group in their structure and are effective for chronic pain management, are able to block NMDAR by the mechanism called “trapping” [19]. Trapping blockers do not prevent channel closure and agonist dissociation. After channel closure, such blockers become trapped in the closed state and cannot leave the channel until it opens again.

**Figure 2:**
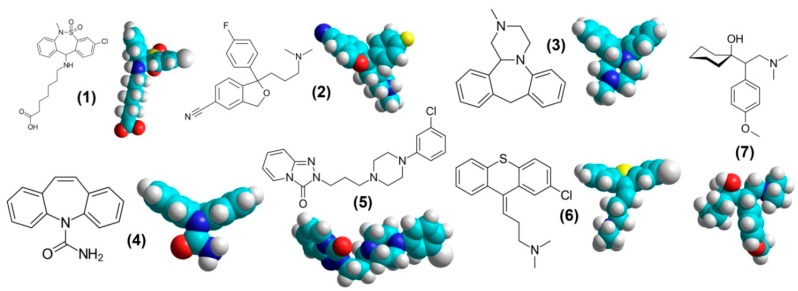
The structures of the psychotropic drugs studied: (**1**) tianeptine; (**2**) citalopram; (**3**) mianserin; (**4**) carbamazepine; (**5**) trazodone; (**6**) chlorprothixene; (**7**) venlafaxine.

However, there were mismatches, too. Our own clinical studies at First Pavlov State Medical University have identified antipruritic and/or analgesic efficacy of the following compounds (Figure 2): tianeptine (atypical tricyclic antidepressant), citalopram (selective serotonin reuptake inhibitors (SSRI)), mianserin (tetracyclic antidepressant), carbamazepine (anticonvulsant), trazodone (serotonin antagonist and reuptake inhibitor (SARI)), chlorprothixene (antipsychotic) [9,20,21,22]. Mianserin, carbamazepine and tianeptine contain the V-like group in their 3D-structure (Figure 2), but they are not NMDAR blockers in clinically relevant concentration [23]. Later, we showed that besides the V-like moiety, a drug has to have a positively charged amine group in its structure to block NMDAR [24], and mianserin, carbamazepine and tianeptine do not contain this. Trazodone and citalopram have other configurations of their aromatic rings, and we showed that they have no anti-glutamate activity [19,23]. The anti-glutamate effect of chlorprothixene is unclear; the angle between its aromatic rings is close to 180 degrees, but the configuration is not completely plain. Our own electrophysiological experiments showed that chlorprothixene is able to inhibit NMDAR, but there was only partial trapping (see details in Section 3.6.3).

Regarding analgesic and antipruritic activity of these psychotropics, certainly, there are some limitations in their effectiveness. Firstly, we studied their effect in patients having chronic itch and pain (musculoskeletal and/or headache) associated with chronic renal failure (CRF) and chronic haemodialysis (CH), as well as in patients with diffuse itching accompanied by depression. It is not necessary that the compounds be helpful for other types of pain and pruritus. Secondly, the drugs were not effective for all persons. Some of them were beneficial for patients of a certain sex; some were ineffective for several subjects for unknown reason. Thirdly, the number of patients of First Pavlov State Medical University that we could observe was limited. Summarising the above, we suppose it is necessary to expand the frontiers of using of psychotropic drugs for treatment of chronic pain and pruritus.

In the group of the compounds described above, there are no representatives of TCAs, gabapentinoids or serotonin-norepinephrine reuptake inhibitors (SNRI), which are also widely used for chronic pain management. That is why we have added venlafaxine, a SNRI, to the list of the drugs reviewed (Figure 2). We have not included TCAs and gabapentinoids in our review, since they have already been studied and described extensively, for example in reviews [25,26,27,28]. Briefly, TCAs are the most effective antidepressants for neuropathic pain; however, they could be considered to be dirty drugs that affect multiple targets and have pleiotropic effects. It was shown that TCAs are able to affect adrenergic and opioid receptors, voltage-gated sodium, potassium and calcium channels, and NMDA receptors. The multiple actions of TCAs lead to many side effects, which limits their use. The gabapentinoids gabapentin, pregabalin (both are widely used for management of neuropathic pain, postherpetic neuralgia, diabetic neuropathy, trigeminal neuralgia, and restless legs syndrome) and mirogabalin (recently approved for neuropathic pain and postherpetic neuralgia in Japan) are ligands of the α2δ subunit of voltage-dependent calcium channels. It is supposed that the drugs are able to affect NMDA receptors, sodium channels, and the endogenous opioid system. These drugs should be used judiciously in patients with kidney problems [29].

The purpose of this review is to analyse and correlate the literature data and the results of our own research on analgesic/antipruritic activity and pharmacological profile of the compounds chosen. The information obtained could help to identify relationships between the pharmacological profiles of the drugs and the peculiarities of their analgesic/antipruritic efficacy, to identify “blank spots” and to determine the future direction for the development of effective personalised therapy in patients with pain/itch and mood disorders.

## 2. Targets of Analgesic and Antipruritic Therapy

In the first stage, we collated currently available information about the main receptors that are considered targets for analgesic and antipruritic therapy.

### 2.1. NMDA and AMPA Receptors

The glutamatergic system plays an important role in the perception of sensory information of various modalities. Glutamatergic synapses participate in mediation of nerve impulses associated with the transmission of olfactory, vestibular, visual, tactile and pain signals. Glutamate receptors are present in all structures and regions of the central nervous system (CNS) that are responsible for responding to pain stimulation. The main participants in the transmission of pain impulse by the family of glutamate receptors are NMDAR and α-amino-3-hydroxy-5-methyl-4-isoxazolepropionic acid receptors (AMPAR) [30].

AMPARs are more involved in acute pain processes [31]. At the same time, a large number of experimental and clinical data indicate the key role of NMDA receptors in the pathogenesis of neuropathic pain syndrome [32]. At present, a low-affinity NMDAR channel blocker, memantine, known to effectively suppress inflammatory tonic pain, is widely used in clinical practice. In addition, NMDAR antagonists potentiate the effects of opioid receptor agonists [33]. In models of neuropathic pain, all classes of NMDAR antagonists have analgesic activity: channel blockers [34], competitive antagonists [35], glycine antagonists [36] and polyamine antagonists [37]. It should be noted that NMDAR antagonists block almost all displays of pain, from behavioral changes to reactions of the endocrine and vegetative nervous systems [38]. Introduction of NMDAR antagonists effectively prevents the development of neuropathic hyperalgesia, and repeated administration reduces hyperalgesia in animals with an already developed neuropathic syndrome [39]. There is a case for the cure of neurogenic pain syndrome after subarachnoid administration of 3-(2-Carboxypiperazin-4-yl)propyl-1-phosphonic acid (CPP), which is a competitive antagonist of NMDAR [40].

Investigation of the analgesic effect of NMDAR antagonists is ongoing, and a targeted search for potential blockers among various classes of drugs is being conducted. A randomised, double-blind, placebo-controlled study of l-4-chlorokynurenine, the precursor of 7-chlorokynurenic acid, which is in turn a well-known antagonist of the glycine site of NMDAR, was performed on healthy volunteers [41]. The results of the study revealed its safety and effectiveness in reducing allodynia, plus thermal and mechanical hyperalgesia caused by administration of capsaicin to the volunteers. It was shown that the analgesic effect of pregabalin (anticonvulsant) is associated with modulation of the NMDAR [42,43]. Our own electrophysiological experiments have shown that antidepressants and neuroleptics (amitriptyline, desipramine, atomoxetine, chlorpromazine), which have the analgesic effect, are capable of voltage- and magnesium-dependent blockade of NMDAR and are not active in the inhibition of AMPAR [19].

### 2.2. Sodium Channels

According to their activation mechanism, sodium channels are divided into voltage-dependent (VGSCs or Nav) and ligand-dependent [44]. Voltage-dependent sodium channels play a key role in neurons, ensuring the generation and distribution of action potential in the CNS and peripheral nervous system (PNS) [45]. By subunit composition, there are nine types of voltage-dependent sodium channels: Nav1.1-1.9 [46]. Nerve damage can lead to an increase of activity of sodium channels, which leads to hyperexcitability of neurons and causes neuropathic pain [47]. Thus, Navs are attractive targets for the development of anti-pain therapy. The sodium channel blockers that are currently used for the treatment of neuropathic pain include such classes of drugs as TCA [48], local anesthetics [49], and anticonvulsants [13]. Moreover, plant and animal toxins have been widely tested as potent pain therapeutics. In the paper [50], information was gathered about the toxins inhibiting sodium channels and having an antinociceptive effect. The authors of [51] collected the data on cysteine knot peptides from spider venoms that modulate Nav and performed analysis of the structure–activity relationships of the compounds.

Recent clinical and experimental studies indicate that Nav1.7 plays a crucial role in the hereditary mechanisms of neuropathic pain: mutations in the SCN9A gene were found to be associated with hereditary pain syndromes. The mutations associated with congenital pain sensitivity (CIP) lead to incomplete functioning of the Nav1.7 channels and to the inability to experience pain [52]. Given the role of Nav1.7 in pain management and the fact that expression of Nav1.7 is limited to the PNS, it was suggested that blockers to selectively inhibit Nav1.7 could be the ideal analgesics [53]. Therefore, at present, the efforts of many researchers are aimed at finding and improving such drugs and studying the analgesic action of already known blockers. Thus, the study [54] describes animal testing of new potential Nav1.7 blockers belonging to the class of benzenesulfonamides that have a better ability to penetrate the membrane than their predecessors. In the paper [55], a sulfonamide compound AMG8379 was described which, according to electrophysiological measurements, inhibited Nav1.7 in nanomolar concentrations, and showed an analgesic efficacy in experiments on rodents. In the work [56], the interaction of Navs with the neurotoxin Jingzhaotoxin-34, which is part of the poison of the Chinese tarantula, was investigated. It was found that the toxin had a maximum affinity for Nav1.7 at the resting state, and it showed anti-pain activity in the experiments on animals.

To make the rational design of Nav inhibitors more powerful, the molecular mechanisms of Nav inhibition have been studied extensively. For example, in the paper [57], a model of the eukaryotic sodium channel Nav1.4 was constructed using homologous modelling, and then docking of molecules of local anaesthetics and anticonvulsants, including carbamazepine, into the inside the channel was performed. Using the Monte Carlo minimisation approach, the drug binding site and the binding mode were determined; the structural characteristics of the ligands, which are responsible for the effectiveness of their binding to the sodium channel, were identified. Having applied molecular modelling techniques, the authors of [58] showed that neutral and positively charged ligands bind with different sites of the Nav. The neutral molecules bind in the well-known site on helix 6, and the positively charged drugs interact with an additional site, where their charged group is attracted into the selectivity filter. The researchers assumed that the new information about the mechanism of sodium channel blocking would help to design the subtype-selective sodium channel inhibitors.

### 2.3. Calcium Channels

The term “calcium channels” often means the voltage-dependent type of the channels, which is more common, but it should be mentioned that there are ligand-dependent calcium channels as well [59]. According to their physiological and pharmacological properties, the voltage-dependent calcium channels (VGCCs) are divided into several types: low-threshold T-type (Cav3.1, Cav3.2, Cav3.3) and high-threshold L- (Cav1.1-4), N- (Cav2.2), P/Q- (Cav2.1) and R- (Cav2.3) types [60,61]. All of these five subclasses are present in the CNS and PNS. Regulation of intracellular calcium through the activation of VGCCs plays a role in the release of neurotransmitters, depolarisation of membrane potential, activation and inactivation of enzymes, and regulation of gene expression [62,63]. The large amount of data clearly indicates that VGCCs are involved in the pathogenesis of various diseases, including the transmission of pain signals [63,64].

N-type calcium channels are the most attractive target for analgesic drugs [65]. These channels are located in the dorsal root ganglia, on the presynaptic terminals, where afferent nerve fibers form synapses with postsynaptic neurons of the posterior horn of spinal cord [66,67]. Changes of the biophysical properties and increased expression of this type of VGCCs under pathological conditions increase the release of synaptic vesicles with pain transmitters, such as glutamate, substance P, and calcitonin gene-related peptide (CGRP), resulting in pain. The role of N-type VGCCs in neuropathic pain is supported by the data indicating that N-type calcium channel antagonists can block tactile allodynia caused by nerve damage [68] and neural responses of the posterior horn of spinal cord [69]. It was shown that blocking N-type VGCCs at the level of the spinal cord and sensory neurons inhibits the release of pain-inducing peptides such as substance P, CGRP, and the excitatory neurotransmitter glutamate [70,71]. N-type VGCCs blockers pregabalin, gabapentine and ziconotide are widely used in clinics for pain therapy [72]. Interesting details of the mechanism of analgesic action of gabapentinoids were described in [73]. It is known that α2δ-1, a voltage-activated Ca2+ channel subunit, is a binding site of gabapentinoids. The authors showed that α2δ-1 forms a heteromeric complex with NMDARs in rodent and human spinal cords, and gabapentinoids reduce neuropathic pain by inhibiting forward trafficking of α2δ-1-NMDAR complexes.

Analysis of the data reported in the literature in recent years revealed no clear breakthroughs in the development of new analgesic drugs among calcium channel blockers. Recently, the attention of researchers has focused on T-type calcium channels. T-type VGCCs are found in the primary afferent neurons of the spinal ganglia and in the free nerve endings. They contribute to the initiation of action potential in these sites by reducing the activation threshold [74]. Intensified T-type activity of VGCCs contributes to the development of pain by stimulating the increase of synaptic excitation [75]. The paper [76] describes the compound KYS-05090S, which is a T-type calcium channel blocker. Electrophysiological measurements showed that the drug can inhibit the receptors in low micromolar concentration, and its anti-pain effect was shown in animal models of neuropathic pain (model of the Spared Nerve Injury, SNI). In the work [77], 14 diphenyl-methyl-piperazine derivatives were synthesised and their ability to inhibit T-type calcium channels was tested. Based on the results of the testing, a potential blocker was selected that inhibits T-type channels in the half maximal inhibitory concentration (IC_50_) equal to 4 µM, but does not block L- and N-type varieties. The antinociceptive effect of this compound was shown in rodents with formalin-induced inflammatory pain. In addition to the synthesis and testing of new drugs that can inhibit T-type VGCCs, such compounds are sought in nutraceuticals, which are food components having therapeutic properties. Thus, in the work [78] it was shown that the extract of lavender and rosemary, which includes linalool and rosmarinic acid, can have a modulating effect on T-type calcium channels expressed in the HEK293T cell line.

Despite successful in vitro experiments and preclinical testing of new calcium channel blockers, the results of their clinical trials are controversial. For example, a multicentre, double-blind, controlled and randomised trial with ethosuximide, an anticonvulsant and a T-type channel blocker, was performed recently in 114 patients with non-diabetic peripheral neuropathic pain [79]. Earlier, ethosuximide was shown to have analgesic effects in several chronic pain models. The clinical trial revealed the high number of adverse events in the active treatment group where the drug failed to reduce total pain and showed a poor tolerance.

### 2.4. GABA(A)-Receptors

Another well-known participant of nociceptive transmission and, therefore, a possible target for the analgesics, is the A-type gamma-aminobutyric acid receptor (GABAAR). It is a ligand-dependent ion (chlorine) channel in chemical synapses of the nervous system that inhibits the transmission of nervous excitation and is controlled by gamma-aminobutyric acid (GABA) [80]. The structure of the receptor is a pentamer and consists of three types of subunits in various combinations: α (6 subtypes), β (3 subtypes) and γ (3 subtypes) [81].

There is evidence indicating that neuropathic pain can be controlled by modulating the GABAAR. It was shown that spinal administration of the GABAAR antagonist bicculine causes tactile allodynia and thermal hyperalgesia in rats [82]. In contrast, small doses of the GABAAR agonist muscimol inhibited tactile allodynia in rats [83]. The ability of muscimol to reduce neuropathic pain is described in [84] too; moreover, based on observation of the behavior of rodents, the authors concluded that the introduction of muscimol reduced not only pain, but itching as well. Synthetic compound L-838,417 is an agonist of the α1, α2, and α3 subunits and an antagonist of the α5 subunit of the GABAAR; it was shown that this drug has the ability to reduce neuropathic pain in animal models without an addictive effect [85]. The important role of the GABAAR, especially its α2 and α3 subunits, in the mechanism of neuropathic pain is also shown in other studies: the partial agonist of the α2 and α3 subunits of the GABAAR TPA023 has antinociceptive efficacy [86]; the compounds HZ166 [87,88] and SL651498 [89] have similar properties.

Interaction with the GABAAR is described not only for experimental drugs, but also for several clinically approved psychotropic drugs. Electrophysiological experiments in rat hippocampal slices showed that the antidepressants imipramine, fluoxetine and trazodone influenced the interaction of GABAAR with its antagonist pentylenetetrazole [90]. The authors concluded that these antidepressants enhance the activity of the GABAAR complex by an unexplained mechanism. It was shown that carbamazepine and oxcarbazepine at clinically relevant concentrations potentiate the GABAAR [91]. At the same time, according to our data, carbamazepine, imipramine and trazodone are effective in management of neuropathic pain and itching syndromes [15,20,21,22].

The role of the GABAAR in the transmission of different types of pain is currently being studied intensively. For example, the authors of [92] investigated the role of peripheral GABAAR signalling on pain hypersensitivity by using the Freund’s adjuvant (CFA)-induced inflammatory pain model in mice. It was shown that the nociceptive behaviour and mechanical hypersensitivity in CFA-induced mice was significantly inhibited by GABAAR antagonists picrotoxin (a non-competitive GABAAR inhibitor) and bicuculline (a competitive inhibitor unable to cross the blood-brain barrier). Moreover, intraplantar injection of an anti-GABA antibody attenuated CFA-induced mechanical hypersensitivity, and intraplantar application of a selective positive allosteric modulator of GABAAR induced mechanical hypersensitivity in the control group of mice. The authors proposed that peripheral GABAAR signalling in inflamed tissues is a novel potential therapeutic target for alleviating persistent inflammatory pain.

In the paper [93], the α6 subunit of the GABAARs located in trigeminal ganglia was tested as a possible target for management of trigeminal neuropathic pain. The effect of two pyrazoloquinolinone compounds, DK-I-56-1 and DK-I-87-1 was studied, and one of them, DK-I-56-1, was shown to have a therapeutic potential. Electrophysiological experiments demonstrated that the drug enhanced the currents through recombinant rat α6β3γ2, α6β3δ and α6β3 GABAA receptors, whereas it was inactive on most GABAA receptor subtypes containing other α subunits. In the rat chronic constriction injury (CCI) model of neuropathic pain, DK-I-56-1 displayed the antinociceptive effect.

The authors of [94] investigated the participation of spinal α5-GABAA receptors in the reserpine-induced fibromyalgia-type pain in rats. The analgesic effect of L-655,708 (selective α5-GABAA receptor inverse agonist) and (TB 21007, selective α5-GABAA receptor inverse agonist) was tested in reserpine-treated rats. It was shown that both drugs decreased tactile allodynia and muscle hyperalgesia in female rats, and their effect on male animals was lower. In naïve rats, intrathecal administration of L-655,708 and TB 21,007 produced sex-dependent tactile allodynia and muscle hyperalgesia. The effect of L-655,708 was prevented or reversed by L-838,417, a partial agonist of α5- GABAA receptors, which proved that spinal α5-GABAA receptors participate in L-655,708-induced antiallodynic activity in female rats. The researcher concluded that spinal α5-GABAA receptors play a sex-dependent antiallodynic and antihyperalgesic role in naïve rats, suggesting that α5-GABAA receptors inversing agonists may be useful in women with fibromyalgia.

And finally, using cryo-electron microscopy, Masiulis and co-authors recently obtained the 3D-structures of the full-length human α1β3γ2L GABAA receptor and its complexes with the channel-blocker picrotoxin, the competitive antagonist bicuculline, the agonist GABA, and the classical benzodiazepines alprazolam and diazepam [95]. The obtained structures will help to study the details of the mechanism of action of the GABAA receptor by molecular modelling methods and perform a rational design of the analgesic drugs targeting the receptor.

### 2.5. Opioid Receptors

Opioid receptors were identified in the early 70s of the 20th century as the receptors that bind morphine-like substances [96]. Opioid receptors belong to the family of G-protein coupled receptors (GPCRs) [97]. Currently, opioid receptors are divided into three main subtypes: μ- (mu), δ- (delta) and κ- (kappa) [98]. Within the body, the main function of opioid receptors is participation in regulation of pain. In response to pain stimulus, endogenous opioid ligands (opioid peptides) are produced. Their binding with opioid receptors leads to the closure of voltage-dependent calcium channels and to efflux of potassium ions from the cell, hyperpolarisation of membrane potential and termination of nerve impulse transmission [99]. For this reason, opioid receptor agonists (opioids, in other words) remain among the most commonly used analgesic drugs [100,101,102]. Side effects of opioids can include itching, sedation, nausea, respiratory depression, constipation and euphoria, and with continuous use, tolerance and dependence develop, leading to a withdrawal syndrome [103].

Currently, the efforts of researchers are aimed at the development of the opioid receptor agonists that would cause the maximum analgesic effect with minimal side effects [104,105]. Tiwari and co-authors investigated the efficacy and safety of demorphin-[D-Arg2, Lys4]-(1-4)-amide (DALDA), which is an agonist of μ-opioid receptors of the PNS, in animal models of neuropathic pain [106]. It has been shown that the drug was effective for reducing thermal allodynia, while not causing any changes in locomotion and motor activity of the experimental rodents, which indicates the absence of the effect of the drug on the CNS. The authors of [107] investigated the effect of the κ-opioid receptor agonist U50,488 on bone pain caused by breast cancer. Clinical studies revealed a higher efficacy of U50,488 compared with the traditional opioids. It was shown that the use of U50,488 did not lead to bone loss or proliferation of cancer cells.

Currently, the efforts of researchers are aimed at the development of the opioid receptor agonists that would cause the maximum analgesic effect with minimal side effects [104,105]. Tiwari and co-authors investigated the efficacy and safety of demorphin-[D-Arg2, Lys4]-(1-4)-amide (DALDA), which is an agonist of μ-opioid receptors of the PNS, in animal models of neuropathic pain [106]. It has been shown that the drug was effective for reducing thermal allodynia, while not causing any changes in locomotion and motor activity of the experimental rodents, which indicates the absence of the effect of the drug on the CNS. The authors of [107] investigated the effect of the κ-opioid receptor agonist U50,488 on bone pain caused by breast cancer. Clinical studies revealed a higher efficacy of U50,488 compared with the traditional opioids. It was shown that the use of U50,488 did not lead to bone loss or proliferation of cancer cells.

In the work [108], the efficacy and safety of mixed diarylmethylpiperazine opioid receptor agonists were investigated in vitro and in vivo (in rats and macaques). The authors tried to find a drug with such ratio of affinities for the δ-, κ-, and μ-opioid receptors, that the drug would be as effective and safe as possible. According to the data obtained, the drugs with the proportion of affinities δ > μ ~ κ have the maximum analgesic effect and cause the least side effects associated with addiction and difficulty in breathing.

In the paper [109], the analgesic action and side effects of three peptidomimetics AAH8, AMB46, and AMB47, which are agonists of μ-opioid receptors and antagonists of δ-opioid receptors, were tested in animal models using the warm water tail withdrawal test. Morphine was used as the reference drug. According to the data obtained, all the drugs produced a strong analgesic effect. After repeated administration, a drug tolerance developed to morphine and AMB46. Significantly fewer signs of withdrawal are observed in mice that receive repeated AAH8. Drug dependence symptoms were not observed for AAH8. The authors concluded that, although AAH8 may be an alternative to classical opioids, a combination of a μ-receptor agonist with a δ-receptor antagonist is not sufficient to prevent the development of tolerance or physical dependence.

In the work [110], the analgesic and side effects of spiradoline (an agonist of κ-opioid receptors) and of its 1:10, 1:3, 1:1, and 3:1 mixtures with morphine and etorphine (agonists of μ-opioid receptors) were studied in animal models. According to the data obtained, the mixtures with greater ratio of μ to κ agonist have more than an additive antinociceptive effect. The authors concluded that κ:μ mixtures might have therapeutic potential for treating pain, enhancing potency of each other, while reducing or avoiding some μ receptor-mediated adverse effects.

An interesting solution to the problem of side effects of opioid receptor agonists was proposed in [111]. The authors suggested that pH-dependent agonists should be used for pain management, and their pKa should be close to the pH of injured tissue. In this case, the drug would have analgesic action without side effects. The researches verified their hypothesis by testing the designed drug in animal models of inflammatory, postoperative, neuropathic, and abdominal pain.

### 2.6. Cannabinoid Receptors

Cannabinoid receptors are a class of cellular receptors belonging to the GPCR superfamily [112]. The receptors bind the endocannabinoid ligands (anandamide and 2-arachidonoylglycerol) and exogenous ligands (cannabinoids themselves and their synthetic analogues). The endocannabinoid system is involved in a variety of physiological processes, including appetite, pain, mood, and memory [113]. Currently, two types of mammalian cannabinoid receptors are known: CB1 (in the CNS and PNS) and CB2 (mainly in immunocompetent and hematopoietic cells) [114].

The analgesic effect of cannabinoid receptor modulators is being actively studied in animal models. The authors of [115] investigated the analgesic effect of celastrol, a substance contained in the root of *Tripterygium wilfordii* and used in traditional Chinese medicine for inflammatory disorders. In mice, inflammatory and neuropathic pain was induced by injection of carrageenan and by SNI, respectively. The intraperitoneal injection of celastrol relieved the swelling and allodynia caused by carrageenan. In the animals with neuropathic pain, celastrol reduced mechanical hyperalgesia. The anti-pain effect of celastrol was abolished by the introduction of an antagonist of CB2. The authors concluded that the analgesic effect of celastrol is related to its ability to activate CB2, and the cannabinoid system can be a good target for anti-pain therapy. In the paper [116], diabetes and subsequent diabetic neuropathy, resulting in tactile allodynia and thermal hyperalgesia, was induced in mice by daily administration of streptozocin. Then, the animals were administered agonists and antagonists of CB1 and CB2. The results of the experiment showed the effectiveness of agonists of both types of cannabinoid receptors for relief of syndromes of neuropathic pain caused by diabetes, but, at the same time, unexpectedly, CB1 and CB2 antagonists had no pronociceptive effect. The antinociceptive effect of the mixed CB1 and CB2 agonist CP55,940 was also demonstrated in mice with chemotherapy-induced neuropathic pain [117].

Despite a large body of evidence having demonstrated the antinociceptive action of CB1 receptor agonists, opposite results were obtained in the paper [118]. Analgesic action of hemopressin and CB1 receptor antagonist, SR141716, was studied in animal models. The authors showed that hemopressin behaves as a CB1 inverse agonist. At the same time, hemopressin and SR141716 had antinociceptive effect in different models of pain.

There were attempts of clinical trials of CB1 and CB2 modulators for the treatment of neuropathic pain. In a double-blind, placebo-controlled study in cancer patients suffering from neuropathic pain caused by chemotherapy, the efficacy of the cannabinoid-containing drug nabiximols was shown to relieve pain symptoms [119]. It is interesting that nabiximols is a combination of two compounds, tetrahydrocannabinol, which is a partial agonist of CB1 and CB2, and cannabidiol, which is an indirect antagonist of these receptors.

Thereby, modulation of chronic pain through the cannabinoid system is probably more complicated than direct activation or inhibition of cannabinoid receptors.

### 2.7. 5-HT7 Receptors

The 5-HT7 receptors are one of the subtypes of serotonin receptors belonging to the family of GPCRs on the cell membrane. It is activated by the neurotransmitter serotonin [120]. The 5-HT7 receptors are involved in the regulation of body temperature, circadian rhythm, sleep, learning and memory processes [121]. There is also evidence that the 5-HT7 receptors may be involved in the regulation of mood and emotions, which makes them an important target in the treatment of depression and in the development of new antidepressants [122,123].

Data have been accumulated indicating that the 5-HT7 receptors are involved in the transmission of the pain signal. Brenchat and co-authors caused mechanical hypersensitivity in mice using capsaicin [124]. They revealed that the systematic administration of the 5-HT7 receptor agonists had an antinociceptive effect, which was suppressed by administration of the 5-HT7 receptor antagonists. The analgesic effect remained unchanged after administration of the 5-HT1A receptor antagonists, which suggests that it is the 5-HT7 receptor subtype that is involved in the control of pain in conditions involving central sensitisation. The same group of researchers showed that the 5-HT7 receptor agonists could reduce mechanical hypersensitivity and thermal hyperalgesia in mice caused by nerve damage without causing addiction [125].

Study of the analgesic effect of 5-HT7 receptor agonists is ongoing. It was found that the selective agonists of 5-HT7 receptor LP-44 and LP-211 reduced orofacial pain caused by the introduction of formalin in mice [126]. Since chronic pain and depression often accompany and reinforce each other, and the serotonin receptors are involved in the regulation of mood and emotions, the efforts of many scientists are aimed at finding the agonists that would reduce both pain and depression. A group of Italian and Swiss scientists showed in mice that the 5-HT7 receptor agonist LP-211 affects both the sensory and the emotional component of pain [127]. In the work [128], it was shown that an extract of marine cyanobacteria contains compounds having a high affinity for the 5-HT7 receptors. The extract was tested in mice with SNI-induced pain and depression. It was revealed that administration of the compounds isolated from marine cyanobacteria caused antidepressant and anxiolytic effects in males, but not in females.

The 5-HT7 receptors are of great interest also due to existing data on the relationships between the nervous and humoral systems. Neurotransmitters, such as dopamine and serotonin, regulate the secretion of various cytokines by stimulating the corresponding receptors on the surface of immunocompetent cells, including the peripheral ones. It was shown that in patients with mental disorders, the content of proinflammatory cytokines is increased in the cerebrospinal fluid. Cytokines are known to cause hyperalgesia, reduce pain threshold, sensitise afferent nociceptive neurons, and increase the frequency of discharges in nociceptive Aδ- and C-fibers. All these factors contribute to central sensitisation, which is displayed by secondary hyperalgesia and/or allodynia [129]. For this reason, in our opinion, the 5-HT7 receptors are a very promising target for the treatment of chronic pain associated with depressive and anxiety disorders.

### 2.8. Sodium-Calcium Exchanger

The sodium-calcium exchanger (NCX) is a cell membrane bi-directional transporter. Under normal conditions, it removes calcium out of the cell (direct mode) but can also pump calcium into the cell (reverse mode) under special conditions, such as an increased concentration of intracellular sodium and/or membrane depolarisation [130,131].

The authors of the work [132] showed that the TCAs imipramine, desipramine, amitriptyline and clomipramine (having antinociceptive efficacy) inhibit calcium uptake induced by sodium-calcium exchange, whereas the L-type calcium channel blocker diltiazem (not having analgesic activity) does not.

It was shown that many ion channel blockers are partial inhibitors of the NCX, for example, amiodarone (calcium channel blocker), dronedarone (calcium, potassium, sodium channels blocker), bepridil (calcium channels blocker), sibenzolin (sodium channels blocker) [133]. At the same time, amiodarone was found to reduce heat, cold and tactile allodynia in animal models of neuropathic pain [134].

At present, the NCX is considered to be one of the possible targets of therapeutic effect in the development of new methods of pain therapy [135]. The advantage of this target is that, unlike ion channel blockers with strong side effects, regulation at a more delicate level through the NCX is less traumatic for the body. There is a lot of evidence that NCX inhibitors can be used as the regulators of cell calcium concentration, including for the relief of neuropathic pain syndromes and for neuroprotection [135]. The NCX was found to play a key role in the process of calcium-dependent inactivation of NMDAR [136]. It was revealed that when the NCX was affected by the non-specific and specific inhibitors (lithium and KB-R7943, respectively), a significant increase in calcium-dependent inactivation of NMDARs was observed in the presence of calcium in the extracellular solution, which indicates the significant role of the NCX in controlling ionic conductivity of NMDAR. At the same time, lithium is actively used in the treatment of mental disorders; however, its mechanism of action remains unclear. The data obtained make it possible to consider the possible therapeutic use of KB-R7943 and the NCX inhibitors that are similar to it in the mechanism of action. It was proposed that this effect is determined by a tight functional interaction of NCX and NMDAR molecules because of their co-localisation in membrane lipid rafts [137].

### 2.9. Histamine Receptors

In addition to the targets for analgesic drugs, potential targets for antipruritic compounds are also of great interest. Histamine is one of the most well-known pruritus mediators [138,139]. The histamine receptors belong to the class of GPCRs and bind histamine as the major endogenous ligand [140]. Four types of histamine receptors are known: H1–H4 [141]. The role of the H1, H3, and H4 receptors in the transmission of histamine-induced pruritus has been shown in many studies [142,143,144]. For this reason, histamine receptor antagonists are first-line drugs for itch management [145]. Diphenhydramine discovered in 1943 was the first antihistamine clinically approved in the United States [146]. Since then, the development and improvement of histamine receptor antagonists has been continuing. Among the recent studies, the work [147] should be mentioned. The authors synthesised a series of pyrrolidine amides on the base of phthalazinone and tested in vitro their effectiveness against the H1 receptors and the duration of action. Three compounds from the series turned out to be better in terms of duration of action than azelastine, an antihistamine widely used in medicine. The authors of [148] selected twenty steroid compounds from the database with the highest efficacy against the H3 receptor and, then, by sequential modification of the moieties of their chemical structure, they obtained a new generation of compounds with better affinities. Ko and co-authors used a similar approach, searching for new effective H4 receptor antagonists for the treatment of atopic dermatitis [149]. Using the virtual screening method, they selected forty-two of the most promising compounds from the database of small molecules based on the pharmacophore model. Then, using a combination of in silico and in vitro methods they performed a step-by-step modification of the fragments of their chemical structure and obtained a new compound with the improved affinity and selectivity for the H4 receptor.

However, it is known that antihistamines are ineffective in many cases of chronic itching, which indicates the existence of a histamine-independent mechanism [138]. Therefore, in addition to the histamine receptors, other targets for anti-itch drugs are being studied. In particular, transient receptor potential channels (TRP channels) are of great interest as targets for antipruritic therapy.

### 2.10. TRP Channels

TRP channels are a group of ion channels located mainly in the plasma membrane of many types of animal and human cells. There are seven subfamilies of TRP channels: TRPC (Canonical), TRPV (Vanilloid), TRPM (Melastatin), TRPA (Ankyrin), TRPP (Polycystin), TRPML (Mucolipin), TRPN (No mechanopotential). Most of these channels mediate various sensations, such as the sensation of pain, heat, warm or cold, different types of taste, pressure and vision. The channels have relatively non-selective permeability for cations, including sodium, calcium and magnesium ions. TRP channels are activated and regulated through various stimuli and synthesised in almost all body systems [150].

A large amount of data has been accumulated on the involvement of TRP channels in pain pathways and on studies of modulators of the receptors for the management of neuropathic pain syndromes. This information has been fully presented in recent reviews [151,152]. Currently, TRP channels are attracting the attention of scientists due to the fact that some of them are the targets for the treatment of neuropathic pruritus. Six TRP channels are now firmly associated with itch generation and transduction, and selective drugs targeting TRP channels are promising compounds for the treatment of chronic itching [153]. Thus, in the work [154], the mechanism of action of crotamiton, which is a well-known antipruritic drug, was investigated. By patch-clamp technique in the “whole cell” configuration, it was revealed that crotamiton is a strong inhibitor of TRPV4 channels. In vivo experiments in mice showed that the antipruritic efficacy of crotamiton decreased with the injection of GSK1016790A, which is a selective antagonist of TRPV4 channels. Akiyama and co-authors caused itching with various stimuli in wild-type mice and TRPV4 channels knock-out mice and compared the response [155]. It was found that serotonin-induced pruritus in knock-out mice was less exhibited than in wild-type mice. Prior administration of the TRPV4 channels antagonist reduced itching in wild-type mice. The effect was not observed for histamine-evoked scratching. In contrast, having compared scratching responses evoked by histaminergic pruritogens in TRPV4 knock-out and wild type mice, the authors of [156] demonstrated a significant role of TRPV4 channels in mediating histaminergic itch. In the paper [157], it was shown that knockdown of TRPV4 expression in keratinocytes and dermal macrophages reduces allergic and non-allergic chronic itch in mice, respectively. TRPV4 expression in skin biopsies from patients with chronic idiopathic pruritus was significantly more intensive than in skin from healthy control subjects. The authors of all the listed papers agree that TRPV4 channels are a perspective target for the development of antipruritus drugs.

In our review, we couldn’t cover all possible participants of the pain/itch signal transmission. There are many other macromolecules that are considered to be targets of analgesic/antipruritic therapy: adenosine receptors [158], P2X7 receptors [159], noradrenaline and dopamine systems [160], potassium channels [161], SNAP-25, which is a protein participating in docking of synaptic vesicles with the presynaptic neuron membrane [162], etc. These targets definitely require additional consideration in the future.

## 3. Psychotropic Drugs for Management of Pain and Itching Syndromes and Their Interaction with the Targets for Analgesic and Antipruritic Therapy

At the next stage of our review, we searched the data on analgesic and antipruritic efficacy of the psychotropic drugs studied in animal models and in clinical trials. Furthermore, the interaction of the drugs with the listed above targets for analgesic and antipruritic therapy was also considered. The information found is summarised in Table 1 and Table 2.

### 3.1. Tianeptine

#### 3.1.1. Tianeptine. Animal Models

Kim and co-authors studied the effect of tianeptine in a rat model of inflammatory pain caused by intraplantar formalin injections [163]. The pain behaviour was quantified by counting the number of flinches in the injected paw. Tianeptine was administrated intrathecally. The drug reduced the flinching response during both phases of the test and authors concluded that tianeptine is effective in reducing inflammatory pain.

In the work [164], mechanical allodynia was evoked in rats by L5 and L6 SNL and mechanical sensitivity was tested by von Frey filaments. The authors showed a significantly increased paw withdrawal threshold in operated rats after preventive intrathecal tianeptine administration. Its preventive intraperitoneal administration had no effect on the severity of mechanical allodynia. Nevertheless, intraperitoneal pretreatment of tianeptine potentiated the antiallodynic effects of subsequently administered tianeptine. The authors concluded that tianeptine can be used in the clinic for the treatment of neuropathic pain.

One more group of Korean scientists studied the effects of tianeptine on mechanical allodynia in rats [165]. Tactile allodynia in rodents in this study was caused in two ways: by ligating the L5 spinal nerve and by chemotherapy (systematic vincristine injections). The severity of allodynia was also determined using von Frey filaments. Tianeptine was administered intraperitoneally after the appearance of mechanical allodynia in rats. For both models of neuropathic pain, it was shown that tianeptine increases the paw withdrawn threshold and could be effective for the relief of neuropathic pain.

The authors of the work [166] studied the effect of tianeptine on fibromyalgia symptoms in mice. Mice were subjected to chronic stress (daily immobilisation, alternating with cold), which caused symptoms of fibromyalgia. Tianeptine was administered intraperitoneally daily throughout the entire period of stress. Using several behavioural tests and measuring corticosterone levels, it was shown that tianeptine reduces the severity of fibromyalgia symptoms.

Thus, tianeptine was effective for reducing the symptoms of neuropathic pain in several experiments on rodents (Table 1). As for the itch, we were unable to find published papers devoted to the study of the antipruritic effect of tianeptine on animals.

#### 3.1.2. Tianeptine. Clinical Studies

Analysis of the literature did not reveal published work on the clinical trials of tianeptine as an analgesic and/or an antipruritic drug. As for our own data, at the Center for Haemodialysis in the First Pavlov State Medical University of St. Petersburg, we studied the effect of antidepressants tianeptine, citalopram and mianserin on chronic pain and itching in patients with chronic renal failure (CRF) who were on chronic haemodialysis (CH) [9]. The study included 57 patients (35 males and 22 females) with CRF and pain (chronic musculoskeletal pain and/or chronic headache) and itch syndromes. The antidepressants were prescribed randomly in three groups. Analgesics, antihistamines and sedatives were completely excluded from active therapy seven days before the start of treatment with the antidepressants. In our studies there were no refusals of therapy, the severity of adverse effects was negligible. The comparison group included 20 patients with CRF and chronic pain/itch syndrome, but not receiving the antidepressants (but still receiving their usual analgesic and antipruritic therapy). The group taking tianeptine included 20 patients. The daily dose was 37.5 mg. After 6 weeks of treatment, the pain decreased on average by 52% in men taking tianeptine, and by 51% in women. There was no significant reduction in pain in the control group (Table 3). As for itching, after 6 weeks of tianeptine administration, itching decreased on average by 38% in men, and by 20% in women. There was no significant change in itch intensity in the comparison group (Table 3).

Thus, in our clinical studies tianeptine has been shown to be effective in reducing neuropathic pain and itching in patients with CRF. As an analgesic, the drug was equally effective for both men and women, and as an antipruritic agent, it turned out to be more effective for men (Table 1 and Table 3). In our studies, there were no refusals of tianeptine therapy; however, neurologic, cardiovascular, and gastrointestinal side effects are commonly reported in literature and there are cases reported on fatal intoxication connected with tianeptine [167].

#### 3.1.3. Tianeptine. Interaction with the Receptors

Our own research has shown that tianeptine blocks neither NMDAR nor AMPAR [23]. Nevertheless, there are data indicating that tianeptine affects ion current through NMDAR and AMPAR not directly, but by participating in a signalling cascade, which results in the phosphorylation of these receptors and modulation of their permeability [168].

In the paper [169], with the help of a radioactively labelled ligand, it was shown in cell cultures from humans and mice that tianeptine is a high-affinity μ-opioid receptor agonist, a medium-affinity full δ-opioid receptor agonist, and is not a κ-opioid receptor agonist. The authors of [170] caused visceral pain in rats by colorectal distension. The antinociceptive effect of tianeptine disappeared after injection of naloxone (opioid receptor antagonist). The authors concluded that tianeptine is an opioid receptor agonist, which may be associated with its analgesic efficacy.

Lin and co-authors showed involvement of the 5-HT7 receptors in the antinociceptive effect of tianeptine [171]. The rodents with allodynia caused by SNL were injected intrathecally with tianeptine both in the absence and presence of SB-269970, which is an antagonist of the 5-HT7 receptors. The authors showed that tianeptine had an analgesic effect that was suppressed by SB-269970.

Several ex vivo experiments showed that tianeptine does not bind at clinically relevant concentrations to calcium channels, glutamate receptors, GABA(B) receptors, and histamine receptors [172]. GABAAR were not studied, but we have supposed that tianeptine probably does not affect them either.

Published data does not show the effect of tianeptine on voltage-dependent sodium channels, cannabinoid receptors, the NCX, TRP channels. The information on tianeptine pharmacological profile is summarised in Table 2.

### 3.2. Citalopram

#### 3.2.1. Citalopram. Animal Models

The authors of the work [173] studied the analgesic effect of selective serotonin reuptake inhibitors in two animal pain models: (1) the hot plate test, in which an animal was placed on a pad with certain temperatures (from 42 °C to 53 °C) and measuring times before the nociceptive behavior; (2) intraperitoneal injection of phenylbenzoquinone, which caused a nociceptive response–abdominal writhing as a result of nociceptive irritation of visceral receptors. It was shown that SSRIs, including citalopram, were more effective in pain models with the hot plate test. The authors suggested that this may be due to the fact that in the hot plate test both supraspinal mechanisms (emotional perception) and spinal reflexes are activated. Thus, it seems that citalopram and other SSRIs are supraspinal analgesics: painkillers that promote the release of prolactin, which provides euphoria and distract attention from pain.

The opposite result is described in [174]. The authors studied the analgesic effect of various antidepressants, including citalopram, in rat models of acute, persistent and neuropathic pain. Acute pain was evoked by a hot plate, chronic pain was caused by intraplantar formalin injection, and neuropathic pain was caused by chronic constriction injury (CCI). In this work, citalopram was not effective in the hot plate test, but showed an antinociceptive effect in the second phase of the formalin test, and also reduced thermal hypersensitivity in the CCI model. The researchers did not come to any conclusion regarding citalopram, indicating that there are limits to its use, and this drug, along with other SSRIs, requires further study.

The drug was effective for reducing the symptoms of neuropathic pain in some experiments in rodents (Table 1). As for the itch, we could not find any published papers on testing the antipruritic effect of citalopram in animals.

#### 3.2.2. Citalopram. Clinical Studies

The authors of the work [175] performed a double-blind study of the analgesic action of citalopram in patients with diabetic neuropathy. The patients diagnosed with diabetes had one or more symptoms: pain, paresthesia, dysesthesia and hypoesthesia; and the symptoms of peripheral neuropathy: decreased sensitivity, strength, or tendon reflexes. In the experiment, the patients were randomly divided into two groups (taking citalopram and placebo), not being informed which group they were in. During the first week of the study, the data on their condition were gathered, 3 weeks of therapy followed, then a pause to remove the metabolites was taken; again the patients were divided into two groups, and another 3 weeks of therapy followed. This study showed that citalopram caused a slight relief of symptoms of chronic diabetic neuropathy. The effect of citalopram was slighter than the effect of TCAs, but citalopram was better tolerated by the patients.

Nørregaard and co-authors [176] in a double-blind study tested the effect of citalopram on patients with fibromyalgia. Over 8 weeks, one group of patients took the drug (n = 22) and another group (n = 22) took a placebo. According to the results obtained, citalopram did not affect the patients with fibromyalgia.

In 1996, the authors of [177] investigated the effects of amitriptyline and citalopram on 40 patients with chronic tension headaches. A double-blind, placebo-controlled study was performed. The intensity and duration of headache were recorded during 4 weeks before, then 8 weeks of therapy; there was a 2-week pause to remove the metabolites, again 8 weeks of therapy, a 2-week pause, and final 8 weeks of therapy followed. A slight reduction of pain (“no” rather than “yes”) was obtained. The conclusion of the research was that citalopram is not suitable for the treatment of tension headaches.

The authors of the work [178] also investigated the effect of citalopram on female patients with fibromyalgia in a double-blind, placebo-controlled study. After 2 months of treatment, a significant decrease of pain was observed, but at the end of the test period (4 months) the level of pain increased again. A slight decrease was also observed in the placebo group. The difference between the groups receiving citalopram and placebo did not achieve statistical significance.

Aragona and co-authors [179] investigated patients with various types of psychosomatic pain: headache, fibromyalgia, arthritis pain, and lower back pain. Patients were given citalopram and reboxetine (a noradrenaline reuptake inhibitor). About 30% of patients gave up therapy because of severe side effects. However, it was shown that citalopram statistically significantly reduced psychosomatic pain, whereas reboxetine did not. Based on this result, the authors concluded that the antidepressant and analgesic effects of citalopram have different mechanisms.

In the work [180], the effect of citalopram and paroxetine (SSRI as well) on patients with diabetic neuropathy was investigated. A small but statistically significant analgesic effect was shown. The authors concluded that SSRIs are not the first-line drugs, but since they have fewer side effects than TCAs and anticonvulsants, therapists may prescribe them for neuropathic pain management.

The authors of [181] studied the effects of escitalopram (S-enantiomer of citalopram) on patients suffering from moderate severe psoriasis accompanied by depression and/or anxiety. Patients (n = 38) taking antagonists of tumor necrosis factor (these drugs are widely used as agents for the treatment of psoriasis), were divided into 2 groups. In the first group, escitalopram was added to the main set of drugs. The second group took only the main set of drugs. It was shown that patients of the first group had improved depression symptoms, and decreased both anxiety and depression. After using escitalopram (the authors did not indicate the duration of therapy), itching intensity decreased, but then began increasing again. The authors did not make certain conclusions about the effectiveness of citalopram for the treatment of pruritus, suggesting that the relief effect may be related to the general antidepressant and anti-anxiety effects of the antidepressant.

In our study (the design of the experiment is described in Section 3.1.2) in patients with CRF, citalopram had the weakest analgesic and antipruritic effect among three studied antidepressants (citalopram, tianeptine, mianserin). In the group of 20 patients taking this drug in a dosage of 20 mg per day, citalopram reduced the average pain intensity by 18% in men, and by 22% in women (Table 3). Based on the comparison of psychosomatic indicators and pain intensity before and after therapy, we concluded that the ability of citalopram to manage pain was due to its effect on the emotional component of chronic pain [9]. As for antipruritic effect of citalopram, after 6 weeks of therapy, itching decreased on average by 12% in men and by 22% in women (Table 3). Thus, the use of citalopram led to a slight relief of itching, which is probably caused just by the antidepressant and anti-anxiety effects of the drug and the improvement of mood of the patients.

Thus, on the whole, effectiveness of citalopram in reducing chronic pain and itching is absent or weak (Table 1). According to most studies, including our trial, if citalopram still has slight analgesic and antipruritic effect, it is due to its influence on the emotional component of pain/itch. Regarding this observation, it is interesting to notice that in our study citalopram was a little more effective for women, especially in itch treatment. Other researchers did not distinguish patients by sex in their papers except [178] who studied women only.

In the described trials, there were withdrawals and dropouts due to the following side effects: nausea, vomiting, epileptic seizures, tachycardia, dizziness, gastric upset, diarrhea, vertigo [175,176,177,178,179,180] (Table 1).

#### 3.2.3. Citalopram. Interaction with the Receptors

The analysis of the data reported in the literature has not revealed any studies on anti-glutamatergic effect of citalopram. In our study, we showed in isolated rat brain neurons using the patch-clamp technique that citalopram is almost ineffective as an inhibitor of NMDARs (IC_50_ = 180 μM) and not at all effective as an AMPAR inhibitor (IC_50_ > 300 μM) [19].

The authors of [182] using the patch-clamp technique, showed in HEK293 cells that citalopram can inhibit Nav1.8 with IC_50_ = 100 µM. The researchers concluded that the analgesic effect of citalopram and other SSRIs may be partially related to the blockade of sodium channels.

With the help of electrophysiological approaches, the ability of citalopram to inhibit L-type calcium channels was shown in isolated ventilary cardiomyocytes of guinea pig [183] and in rat cardiomyocyte cell culture [184]. In the work [185], the data was obtained in isolated ventilation myocytes from rats that citalopram inhibits L-type calcium channels with IC_50_ = 64.5 μM. Moreover, citalopram did not compete with the L-type calcium channel blocker diltiazem for binding to the receptor, which points to different binding sites of these drugs.

In the review [186], it is reported with reference to the unpublished data that citalopram does not affect the GABAergic system.

In the study [187], rats were administrated with citalopram for 14 days. Then, in discrete brain regions, the authors studied the binding of [35S]GTPγS (a radioactive G-protein activator) in the presence of the cannabinoid receptor agonist, HU210, or the μ-opioid receptor agonist, [D-Ala2,N–Me– Phe4,Gly5-ol]-enkephalin (DAMGO). It was shown that HU210-induced increase in [35S]GTPγS binding was reduced or abolished (depending on brain regions) after chronic treatment with citalopram. Citalopram had no significant effect on DAMGO-induced [35S]GTPγS binding in the brain regions investigated, with the exception of the medial geniculate nucleus where a modest impairment was observed. The authors concluded that citalopram probably affects the cannabinoid system as an inverse agonist and does not affect the opioid one.

Hedlund and co-authors [122] studied the role of the 5-HT7 receptors in the pathways of depression and the mechanism of the therapeutic action of citalopram in mice. The researchers divided the animals into two groups, wild-type and 5-HT7 receptor knockout. They then caused depression in the mice and injected the following drugs: the 5-HT7 receptor inhibitor SB-269970 and citalopram. SB-269970 differently affected the behavior of wild-type and knockout mice, whereas citalopram showed no statistically significant difference. The authors concluded that citalopram does not interact with the 5-HT7 receptor.

Using radioligand binding assays, it was shown that R-citalopram and citalopram possess moderate affinities for histamine H1 receptors (affinity Ki = 181 ± 5 nanomoles/litre and 283 ± 18 nanomoles/litre, respectively) [188].

We have not found published data on the effect of citalopram on the NCX or TRP channels. The information on citalopram pharmacological profile is summarised in Table 2.

### 3.3. Mianserin

#### 3.3.1. Mianserin. Animal Models

The authors of [189] in their experiments demonstrated that mianserin decreased tolerance and increased the latent period of response to painful stimuli in morphine-tolerant mice. However, without mianserin, the indicators did not change.

In the work [190], the tandem effect of mianserin with morphine and indomethacin/metamizole (nonsteroidal anti-inflammatory drugs) was studied in mice. Two models were used in the experiment: the (1) tail-flick and (2) hot plate tests. The effect of a single dose and daily administration of the drug for 14 days was tested. According to the results, mianserin in a single dose increased the antinociceptive effect of metamizole (in the hot plate and tail-flick tests) and indomethacin (only in the tail-flick test). Mianserin, administered for 14 days together with metamizole, enhanced the anaesthetic effect of the latter (in the hot plate and tail-flick tests). Mianserin, applied for 14 days, increased antinociceptive effect of indomethacin (in the tail-flick test). Mianserin did not alter the analgesic effect of morphine.

In 2015, Üçel and co-authors studied the effects of mianserin on neuropathic pain induced by diabetes in rats using several experimental models [191]. The effect of mianserin on hyperalgesia arising from peripheral diabetic neuropathy was studied using the Randall-Selitto test (paw compression test—mechanical nociceptive stimulus), Hargreaves test (“plantar test”—thermal nociceptive stimulus) and the cold plate test (4 °C—thermal nociceptive stimulus). A dynamic plantar aesthesiometer, which measured the threshold values for mechanical stimuli, was used for allodynia studies. Thermal allodynia was evaluated using the hot plate test (38 °C). It was found that mianserin improved mechanical and thermal hyperalgesia associated with diabetic neuropathy. It also reduced diabetes-associated mechanical and thermal allodynia. The ability of mianserin to decrease diabetic neuropathic pain was comparable to pregabalin. Antihyperalgesic and antiallodynic effects of mianserin disappeared when using α-methyl-para-tyrosine methyl ester (AMPT, catecholamine synthesis inhibitor), phentolamine (non-selective α-adrenoreceptor antagonist), propranolol (non-selective antagonist β-adrenoreceptor) and naloxone. However, these same effects remained in the presence of para-chlorophenylalanine methyl ester (PCPA, serotonin synthesis inhibitor). The results suggest that the positive effect of mianserin in diabetic neuropathic pain is connected with the increase of the level of catecholamines in the synaptic cleft as well as through interaction with adrenergic receptors and opioid receptors.

In the work [192], the authors studied the effects of 5-HT receptor antagonists, including mianserin, on pruritus in rats. The hindlimb scratches were induced by subcutaneous injection of 5-methoxytryptamine (5-MeOT, an agonist of 5-HT receptors). After the introduction of mianserin and other antagonists of 5-HT receptors, potentiation of 5-MeOT-induced hindlimb scratching occurred. The authors suggest that mianserin and other drugs can act not only as antagonists of 5-HT receptors, but also as agonists of the receptors mediating hindlimb scratching.

Thus, in experiments in rodents, the drug showed its antinociceptive effect in some models of neuropathic pain (Table 1). The effect of mianserin on itching in animal models was described only in one paper and, according to published data, mianserin did not reduce but rather potentiated itching in rats.

#### 3.3.2. Mianserin. Clinical Studies

In 1989, Langemark and co-authors studied the effect of mianserin and TCA clomipramine on chronic idiopathic pain in a group of 182 patients [193]. During a double-blind, placebo-controlled study, the pain intensity was measured after 3 and 6 weeks of therapy. In the patients that were prescribed mianserin, there was a statistically significant reduction of tension-type headache (84 patients), but the effect of mianserin was weaker than clomipramine. Mianserin was ineffective for other types of pain, such as burning mouth, abdominal pain, and lower back pain.

In the paper [194], the authors decided to confirm/refute the theory about chronic idiopathic pain as a form of “masked depression”. In the experiment, the patients were divided into four groups: (1) patients with pain and “masked depression” diagnosed, (2) patients suffering from pain and depression, (3) patients suffering only from pain, (4) patients with only depression. All the subjects took mianserin as a treatment. As a result, mianserin did not have an analgesic effect in any of the groups. The authors suggested that the analgesic effect of mianserin is exhibited in patients with diagnosed and treatable depression of unknown origin.

In 1993, in a double-blind placebo-controlled study, the authors of [195] tested the analgesic effect of mianserin in the following groups: (1) patients with depression without pain, (2) patients with depression with chronic (organic) pain, (3) patients with psychosomatic disorders and signs of depression, (4) patients with chronic pain without depression. After a 12-week experiment, the scientists had not confirmed the analgesic effect of mianserin.

Two groups of patients took part in the study [196]: (1) patients with depression and chronic tension-type headache and (2) patients only with tension-type headache. The control group took a placebo, the second group of patients in the first 4 weeks took mianserin, the next 8-week period they took fluvoxamine (5-HT reuptake inhibitor). According to the results, both drugs had an analgesic effect; however, higher rates of mianserin were in the group of patients suffering from depression and mild tension-type headache, while fluvoxamine showed a better result in the group not suffering from depression. The authors suggested that such effect occurs due to the involvement of serotonergic neurotransmission in the tension-type pain mechanism. The analgesic effect is probably associated with a direct effect on the central nervous system.

Analysis of the literature did not reveal published work on the clinical trials of mianserin as an antipruritic drug.

In our studies [9] (the experiment design is described in Section 3.1.2) in a group of 17 patients taking mianserin at a dosage of 30 mg per day, the average pain intensity decreased by 67% in men and 24% in women. As for antipruritic effect of mianserin, after 6 weeks of therapy, itching decreased, on average, by 73.6% in men and 17% in women (Table 3). Thus, according to our data, for men, mianserin turned out to be the most effective analgesic and antipruritic drug of the three antidepressants studied (tianeptine, citalopram, mianserin). Moreover, in mianserin therapy, differences in efficacy for men and women were most pronounced.

To summarise, the efficacy of mianserin for chronic pain is uncertain. Most studies indicate a lack of analgesic effect or a slight effect on certain pain under certain conditions, e.g., tension headache in patients suffering from depression. Thus, our results seem to contradict those reported in other works. We suppose that this could be explained by sex difference; our trial demonstrated high efficacy of mianserin in men and a low effect in women. The other studies did not distinguish the effect of mianserin in males and females, but there were more women than men in the studied groups in all of them [193,194,195,196]. With regard to side effects, there were withdrawals and dropouts due to the following side effects: lack of appetite, dryness of mouth, thirst, drowsiness, and dullness [194,195,196].

#### 3.3.3. Mianserin. Interaction with the Receptors

In the work [197], the inhibitory effect of several antidepressants, including mianserin, on the current mediated by the NMDARs of frog oocytes was studied by electrophysiological methods. Mianserin did not show any ability to inhibit NMDARs. Our own electrophysiological studies have also shown that mianserin does not have anti-glutamatergic activity [23].

Using the electrophysiological methods (5 Hz train protocol), a group of Hungarian scientists revealed that mianserin can inhibit the voltage-dependent sodium channels Nav1.2 in human HEK-293 cells with IC_50_ = 43 μM [198]. The same group demonstrated that the effectiveness of the inhibition of sodium channels by mianserin is practically independent of pH, and therefore of the protonation state and the total charge of the molecule of this antidepressant [199].

Becker and co-authors studied the muscle relaxant effect of several antidepressants, including mianserin, in rat aortic rings [200]. According to the data obtained, mianserin, imipramine and sertaline had a muscle relaxant effect on the aorta muscles. The authors suggested that this effect was associated with the blocking of voltage-dependent calcium channels. Here it should be noted that, despite the myorelaxant activity of mianserin shown in this work, its effectiveness was several times lower than that of imipramine and sertaline. The authors of [201] also examined the ability of several antidepressants, including mianserin, to inhibit muscle contraction in isolated sections of the ileum of guinea pigs. It was shown that some antidepressants can inhibit muscle contraction through the blocking of voltage-dependent calcium channels, but mianserin did not have an inhibitory effect.

In the work [202], the effects of imipramine, mianserin, and saline on various signalling pathways were studied. The experiments were carried out in rats, which were divided into several groups. For 14 days, each group of animals was injected with an antidepressant and one with saline. Then, the sections of the pyramidal neurons of the hippocampus were taken from the animals and the effects of major neurotransmitters, including GABA, were measured by electrophysiological methods. It was revealed that mianserin did not affect the effects of GABA compared with the control group. The authors concluded that mianserin does not interact with the GABAergic system.

Olianas and co-authors [203] studied the effects of mianserin on opioid receptors. The objects of the study were Chinese hamster ovary cells (CHO) expressing human δ-, ĸ- and µ- opioid receptors, and neurons of mouse hippocampal and striatal cell cultures. Using radioactively labelled [35S]GTPγS, it was shown that mianserin was about 12- and 18-fold more potent in displacing the radioligand bound to the κ-opioid receptor (Ki = 1.7 ± 0.3 µM) than the µ-opioid receptor-1 (Ki = 21 ± 1.2 µM) and δ-opioid receptors (Ki = 30.2 ± 1.9 µM).

The interaction of mianserin with the opioid system has been shown in vivo as well. In the study [204], the antinociceptive effect of mianserin was demonstrated in rodents using the hot plate test. The analgesic effect of mianserin was eliminated by naloxone, an antagonist of opioid receptors. The injection of other specific antagonists of various types of opioid receptors together with mianserin showed that mianserin has the best affinity for µ-, κ1- and κ3-subtypes. The authors of [191] caused diabetes mellitus in rats, and then injected them with mianserin. Using various tests determining different types of nociceptive disorders (mechanical and thermal allodynia, hyperalgesia), the researchers compared the behaviour of the rats treated with mianserin with the control group. It was found that the antinociceptive effect of mianserin may be related to its effect on adrenoreceptors and opioid receptors.

In the study [205], by measuring contractions in isolated segments of guinea pigs’ ileum, it was shown that mianserin is an effective antagonist of the 5HT7 receptors. The authors of [206] studied the molecular and pharmacological characteristics of the 5-HT2a and 5-HT7 receptors in the salivary glands of the blowfly *Calliphora vicina*. The effects of various antidepressants on the receptors were evaluated by measuring the transepithelial potential. It was determined that mianserin was the most effective antagonist of the 5-HT7 receptors among the studied antidepressants.

Using radioligand binding assays, it was shown that mianserin has extremely high affinity for histamine H1 receptor [207,208], and moderate affinity for H4-receptor [209].

No published data was found either on the effect of mianserin or on cannabinoid receptors, the sodium-calcium exchanger, or TRP channels. Information related to the mianserin pharmacological profile is summarised in Table 2.

### 3.4. Carbamazepine

#### 3.4.1. Carbamazepine. Animal Models

The analgesic activity of carbamazepine has been known since the mid-20th century, and continues to be studied. In rats, carbamazepine has been shown to reduce inflammatory hyperalgesia caused by the introduction of brewer’s yeast [210].

In the study [211], thermal and mechanical pain in mice was induced in the hot plate and tail pressure tests, after which the authors tested antinociceptive effect of sodium channel blockers, including carbamazepine. It turned out that the studied drugs were much more effective for relieving thermal pain compared to mechanical pain, which indicates that there are different ways of transmitting these pain signals.

In another research investigation [212], using the writhing test in animal models, the analgesic effect of antidepressants (imipramine (TCA), fluvoxamine (SSRI), and milnacipran (SNRI)) was evaluated with a joint intake of carbamazepine. According to the results, carbamazepine has an antinociceptive effect, and in combined administration with antidepressants, the analgesic effect is enhanced.

The authors of [213], by administering paclitaxel to rodents, simulated neuropathic pain by chemotherapy and afterwards examined the pain relief effect of anticonvulsants: gabapentin, lamotrigine, and carbamazepine. The result of the study showed that carbamazepine had no significant analgesic effect in this type of neuropathic pain. The opposite result was obtained in the work [214]. The analgesic effect of a single and continuous administration of carbamazepine with cisplatin (a component of chemotherapy) on peripheral neuropathic pain in rats was studied. Pain behaviour was evaluated by the “tail-flick” test. According to the data obtained, one-time carbamazepine intake increased the tail-flick time latency, and continuous administration of the drug increased the pain threshold. The authors concluded that carbamazepine can be used in cancer patients to relieve neuropathic pain caused by cisplatin.

In the work [215], neuropathic pain in rats was induced by SCI. The authors then studied the antinociceptive effect of several psychotropic drugs: cannabinoid receptor agonist CP55,940, gabapentin and carbamazepine (anticonvulsants) and amitriptyline (antidepressant). According to the data obtained, carbamazepine was not effective in this type of neuropathic pain.

In the work [216], mechanical allodynia was induced in rats by infraorbital nerve CCI, thus simulating trigeminal neuralgia. Then, the analgesic effect of various drugs was studied: morphine, baclofen, carbamazepine, clomipramine. It turned out that of all the drugs, only carbamazepine and baclofen had the antinociceptive effect, wherein the effect of carbamazepine was stronger.

Thus, in experiments in rodents, the drug showed its analgesic effect in quite a few models of neuropathic pain (Table 1). As for itching, we could not find any published papers testing the antipruritic effect of carbamazepine on animals.

#### 3.4.2. Carbamazepine. Clinical Studies

In neuropathic pain treatment in humans, carbamazepine is used primarily as a first-line drug for trigeminal neuralgia. The first clinical placebo-controlled study proving the effectiveness of carbamazepine for the treatment of pain symptoms in trigeminal neuralgia was conducted in 1966 in 70 patients [217].

In the study [218], 143 patients with trigeminal neuralgia were treated with carbamazepine. The result of the study showed that carbamazepine was effective in relieving pain in more than 50% of the cases without strong side effects and addictive effects over the prolonged treatment.

The last clinical study on carbamazepine and trigeminal neuralgia was presented in 1995 [219]. The authors studied the effect of carbamazepine and phenytoin. The drugs showed similar efficacy (82.35% and 81.81% responders, respectively), whereas combination therapy showed 100% efficacy.

Carbamazepine is also widely used in the treatment of diabetic neuropathy. In a small group of patients (40 patients), a double-blind placebo-controlled study showed the nociceptive efficacy of carbamazepine in patients with the disease [220]. 

A recent study [221] involved 452 patients with painful diabetic neuropathy. Patients took carbamazepine during a 12-week period. After 2 months, the average pain interference score significantly reduced, and the quality of life (QoL scale score) improved.

Antipruritic efficacy of carbamazepine was also studied. The work [222] describes 3 cases of paroxysmal itching in female patients with multiple sclerosis. In two cases out of three, carbamazepine relieved itching. The study [223] on brachioradial pruritus (BRP) in cervical spine diseases mentions a patient with BRP which had relief of itch during the therapy with carbamazepine. The report [224] described a patient with trigeminal trophic syndrome, who suffered from ulceration near the right nostril, which caused facial pain and itching in the nose. Treatment with carbamazepine decreased the symptoms.

In our small trial, 11 patients were studied with chronic kidney disease on haemodialysis taking a course of carbamazepine. Status of the patients was estimated before and after 4 weeks from the beginning of the therapy. Before the medical treatment pains were localised in upper and lower extremities, vertebral region, and coxal bones; durations of pain paroxysm were from 2−3 h to 20 h per a day; the average pain intensity in the group was 4.98 ± 2.37 (VAS); SCORAD index was 18.0 ± 7.5. After carbamazepine treatment, the respective indexes were: VAS—1.64 ± 0.88; SCORAD index—5.1 ± 2.0 [20,21].

Thus, among the seven psychotropics analysed, carbamazepine was the most studied analgesic drug, mainly in trigeminal neuralgia and diabetic neuropathy (Table 1). Moreover, it seems to be the least controversial: all the described clinical trials state its effectiveness confidently. In our studies, carbamazepine also showed the highest analgesic efficacy for patients with CRF. Among the published studies on carbamazepine, the trials [218,221] were the most extensive, with 143 and 452 patients, respectively. It is quite upsetting that the authors did not publish separate results for male and female patients. In our study, carbamazepine turned out to be a little more effective for men (Table 4), but it would be great to confirm or disprove this result in a bigger group of patients. As an antipruritic agent, according to our study, the drug is much more effective in men than in women (Table 4), and again, we cannot compare the result with other papers because a lack of published data. The disadvantage of carbamazepine is that it has a weaker anti-depressive and anxiolytic effect than mianserin and tianeptine. The reduction of depression and anxiety in carbamazepine therapy was by 18.9–31.1% (Table 4), whereas mianserin was the most effective for men (reduction of depression and anxiety by 74.4% and 71.4%, respectively) and tianeptine was the most effective for women (reduction of depression and anxiety by 52.2% and 48.3%, respectively) [9]. With respect to adverse effects, there were withdrawals and dropouts due to the following side effects: giddiness, rash, and sleeplessness [217,218,220] (Table 1).

#### 3.4.3. Carbamazepine. Interaction with the Receptors

The mechanisms of interaction of carbamazepine with the glutamatergic system are not fully understood. Some studies have found that carbamazepine affects the signalling pathways that involve NMDARs [225,226,227]. This data does not exclude the fact that carbamazepine can interact directly with NMDARs. However, our electrophysiological measurements showed that carbamazepine in therapeutically relevant concentrations does not inhibit either NMDARs or AMPARs [23].

The ability of carbamazepine to block voltage-dependent sodium channels has been shown in many papers at the end of the last century [228,229,230,231]. Currently, a lot of studies are focused on determining the mechanism of this blockade. Thus, in [232] it was shown that carbamazepine inhibits Nav1.7 channels by external, but not internal, application in whole-cell recording. In the “inside-out” configuration, carbamazepine inhibits only from the inside of the membrane. A similar result was obtained for the local anaesthetic lidocaine. The data obtained indicated that lidocaine and carbamazepine have similar penetration routes to the binding site, and the binding site itself is located on the inner side of the membrane.

In the work [233] in guinea pig hippocampal slices, a condition of epilepsy was caused using three different agents: low level of magnesium, caffeine, and penicillin. Then paroxysmal depolarisation shifts were measured in sections with and without added carbamazepine. It turned out that the effect of carbamazepine on paroxysmal depolarising changes has the same characteristics as calcium channel blockers. The authors suggested that carbamazepine can block calcium channels. Later on in the work [234], the effects of three anticonvulsants (phenytoin, carbamazepine and gabapentin) on voltage-dependent calcium channels of L-, N- and P-type (high voltage activated calcium channel) were studied in patients with chronic temporal epilepsy. The experiment was performed in patients’ isolated hippocampal neurons using the method of fixing the potential in the “whole-cell” configuration. It was shown that carbamazepine does not interact with calcium channels in therapeutically relevant concentrations.

In the paper [235], it was shown that carbamazepine potentiated the flow of chlorine ions through GABAAR, induced by GABA, in human embryonic kidney cells and in rat cortical neuron cultures with half maximal effective concentration EC_50_ = 24.5 nM. At the same time, carbamazepine affected only the GABAARs containing gamma subunits and not the recombinant receptor containing only alpha and beta subunits. Moreover, benzodiazepines did not affect the effect of carbamazepine, which suggests different binding sites for carbamazepine and benzodiazepines.

There is no data in the literature about the direct effect of carbamazepine on opioid receptors. However, it has been shown that opioid receptor ligands and carbamazepine can enhance the effects of each other. Thus, in the work [236], epileptic seizures were caused by electroshock in mice and the threshold of this seizure was measured. After that, the effect of various anticonvulsants and antagonists of opioid receptors on the threshold was studied. Naloxone, a non-selective opioid receptor antagonist, has been shown to enhance the anticonvulsant action of carbamazepine. In animal experiments [237] and in clinical trials [238] carbamazepine has been shown to increase the analgesic effect of opiates in the treatment of neuropathic pain.

In the work [239] in a streptozotocin-induced diabetic neuropathy model, the analgesic effect of eslicarbazepine acetate, a derivative of carbamazepine, was studied. The drug was effective for the treatment of neuropathic pain, and the cannabinoid receptor antagonists AM251 and AM630 significantly inhibited its antinociceptive effect. The authors came to the conclusion that eslicarbazepine acetate can interact with cannabinoid receptors. It is possible that carbamazepine, close in structure to eslicarbazepine acetate, is also capable of this.

Oláh and co-authors, using mouse fibroblast cell culture expressing rat TRPV1 channels, studied the uptake of radioactive calcium caused by the effect of vanilloids on TRPV1 channels and the effect of various psychotropic compounds on this uptake [240]. It turned out that carbamazepine does not have a significant impact on the operation of TRPV1 channels.

We could not find published data on the effect of carbamazepine on 5-HT7 receptors, the NCX, or histamine receptors. The information on carbamazepine pharmacological profile is summarised in Table 2.

### 3.5. Trazodone

#### 3.5.1. Trazodone. Animal Models

The authors of [241] showed the antinociceptive effect of trazodone in mice in the hot plate test. Moreover, addition of naloxone (opioid receptor antagonist), and metergoline (non-selective serotonin inhibitor), reduced the antinociceptive effect of trazodone. However, lowering of trazodone effectiveness was not observed after the addition of yohimbine (an a2-adrenergic receptor antagonist). Administered with various opioid receptor agonists, trazodone significantly potentiated antinociception. A small (inactive) dose of trazodone potentiated the antinociceptive effect of a selective 5-HT agonist.

Okuda and co-authors induced neuropathic pain in rats by sciatic nerve ligation [242]. Then three experiments were performed: (1) hot plate test; (2) injection of 5,7-dihydroxytryptamine (neurotoxin, used to reduce serotonin in the brain) into the dorsal raphe nucleus and medial raphe nucleus or raphe nucleus magnus; and (3) in the third experiment, the authors tested the sedative effect of trazodone by placing rats in an activity cage. In the experiment with the hot plate, a significant reduction of pain syndrome was found in rats receiving trazodone compared with the control group. In the experiment with 5,7-dihydroxytryptamine, significant differences were also observed between the group taking the drug and the control group, and according to the data obtained, the authors concluded that the neurons of the raphe nucleus magnus are the target for the analgesic action of the drug. The authors did not find a clear sedative effect of the drug. According to their findings, trazodone reduces the painful effect of thermal hyperalgesia and neuropathic pain induced through the descending pathways of serotonergic neurotransmission.

The authors of [243] tested the analgesic effect of trazodone in a formalin test. Before the beginning of the experiment, mice were injected with trazodone intracerebroventricularly and intrathecally. They established the maximum effect of the drug within an hour after the injection; during the subsequent time, the effect decreased. With the introduction of the 5-HT1 receptor antagonist, the analgesic effect of trazodone was reduced, which led the authors to conclude that trazodone is a non-specific 5-HT1 agonist. The authors also suggested that the effect was associated with descending pathways of serotonergic neurotransmission.

Thus, trazodone demonstrated analgesic efficacy in several experiments in rodents (Table 1). As for itching, we could not find any published papers testing the antipruritic effect of trazodone in animals.

#### 3.5.2. Trazodone. Clinical Studies

Wilson and co-authors, in their study [244], found out that trazodone reduced or completely relieved neuropathic pain in patients with type 1 and type 2 diabetes. Moreover, the effect was achieved faster than in treatment with TCAs. The authors suggested that this effect occurs because the drug is a selective serotonin inhibitor.

The authors of the work [245] revealed in a 12-week experiment that trazodone relieved pain in fibromyalgia insignificantly; however, it had a favourable effect on concomitant syndromes such as morning fatigue, general fatigue, etc. The drug showed significant results in restoring normal sleep in patients with fibromyalgia. In the second phase of the experiment, when pregabalin was added to trazodone, there was a large improvement in fibromyalgia symptoms. In conclusion, the authors suggested that trazodone was not suitable as monotherapy for fibromyalgia, but may be prescribed in addition to other drugs.

Similar results were obtained in the work [246] devoted to study of the effectiveness of the combined use of trazodone and pregabalin for relieving the symptoms of fibromyalgia.

Analysis of the literature did not reveal published work on the clinical trials of trazodone as an antipruritic drug.

In our small study, the antipruritic effect of trazodone was checked in 17 patients with anxiety and depressive disorders, which also included diffuse itching in the clinical picture. The examined patients had no pathology of kidney or diabetes. Two patients left therapy because of stomach ache. In the remaining 15 patients, the reduction in the severity of pruritus was gradual and reached its maximum (up to 70%) in 2/3 of patients after 6–10 weeks of therapy. Thus, for the first time, we showed the antipruritic efficacy of trazodone in 2/3 of patients suffering from the syndrome of diffuse itching: the reduction was up to 70% [22].

Thus, in several clinical studies, trazodone has shown its effectiveness in reducing chronic pain (fibromyalgia and diabetic neuropathy) and diffuse itching (Table 1). However, there were withdrawals and dropouts due to the following side effects: dizziness, headache, tachycardia, stomach ache [22,244,245,246].

#### 3.5.3. Trazodone. Interaction with the Receptors

Our electrophysiological measurements showed that the anti-glutamate activity of trazodone is very weak [23].

Using the whole-cell patch-clamp method, the authors of [247] studied the effect of various antidepressants, including trazodone, on the neuronal sodium channels of bovine adrenal chromaffin cells. It was shown that, compared with TCAs, trazodone had no effect on sodium channels: 100 μM of trazodone reduced the sodium current by only 19%.

The opposite result was obtained in [248]. The researchers studied the effect of trazodone on potassium and sodium currents in HEK-293 cells and calcium current in human induced pluripotent stem cell-derived cardiomyocytes (hiPSC-CMs), using the whole-cell patch-clamp method. The results of this study showed that trazodone inhibited the peak amplitude of SCN5A-encoded inward sodium current in a concentration-dependent manner with IC_50_ value of 11.07 ± 0.09 μM and inhibited the Ca2+-specific inward current peak amplitude in a concentration-dependent manner with IC_50_ value of 19.05 ± 0.13 μM.

Kraus and co-authors, using he whole-cell patch-clamp method in rat brain slice, showed that trazodone induced inhibition of T-type calcium-dependent calcium channels Cav 3.1, Cav 3.2 and Cav 3.3 in clinically relevant concentrations (IC_50_ value was 23–45 μM) [249]. The experiment was performed on recombinant human calcium channels. The result showed a weak voltage-dependence of this inhibition. The authors concluded that trazodone inhibits calcium channels at the resting membrane potential.

In the study [90], the rats were injected with antidepressants, including trazodone, during a 7-day period. Afterwards, rodents were injected by pentylenetetrazol (GABAAR antagonist) and beta-carboline-3-carboxylic-acid-methylester (reversible agonist of GABAAR). It was shown that some antidepressants, including trazodone, decrease the effect of pentylenetetrazole and beta-carboline-3-carboxylic-acid-methylester, so the authors concluded that these antidepressants interacted with the GABAergic system.

In the work [241], the molecular mechanism of the antinociceptive effect of trazodone was studied in rodents using the hot plate test. The anti-nociceptive efficacy of trazodone was suppressed by the injection of naloxone (a non-selective antagonist of all opioid receptors), beta-funaltrexamine (a specific antagonist of μ-opioid receptors), naloxonazine (a specific antagonist of μ-opioid receptors). However, the efficacy was not suppressed by the administration of nor-binaltorphamine (a specific κ-opioid receptor antagonist) and naltrindole (a specific δ-opioid receptor antagonist). The authors concluded that the analgesic effect of trazodone may be due to its agonist effect on μ-opioid receptors.

The same study reported that the serotonergic system was also involved in the antinociceptive effect of trazodone; the antinociceptive effect of the drug was inhibited by metergoline, a non-selective serotonin receptor antagonist [241].

The interaction of trazodone with histamine H1 receptors of the human brain frontal cortex was studied using the radioligand binding techniques. The results of the study showed that trazodone could be a potent receptor antagonist with an equilibrium dissociation constant of 350 ± 60 nM [250].

No effects of trazodone on cannabinoid receptors, the NCX, and TRP channels have been reported in the literature. The information on trazodone pharmacological profile is summarised in Table 2.

### 3.6. Chlorprothixene

#### 3.6.1. Chlorprothixene. Animal Models

Surprisingly, the analysis of the data reported in the literature did not reveal any studies on the analgesic and antipruritic effects of chlorprothixene in animal models. However, chlorprothixene is used in many experiments as a component of anaesthesia for animals [251,252].

#### 3.6.2. Chlorprothixene. Clinical Studies

The paper [253] describes the effectiveness of chlorprothixene in patients with meralgia paresthetica at a dosage of 100 mg/day in combination with methylprednisolone sodium succinate and procaine injections.

The clinical trial described in [254] involved 30 patients with postherpetic neuralgia; 5 of them had severe neuralgia, and the remaining 25 had moderately severe neuralgia. For the patients with moderately severe cases, the dosage was 50 mg every 6 h orally. The other 5 patients took the same 50 mg orally and then received a 100 mg injection of chlorprothixene intramuscularly. After taking the drug, 5 patients with severe neuralgia experienced relief in the first 24 h. In the group with moderate severity, total relief was experienced in the 24–72 h after starting the administration.

In 1978, the effect of chlorprothixene on postherpetic neuralgia was tested in two double-blinded studies [255]. The first study showed that only 4 of 17 patients experienced pain relief with a low dosage of chlorprothixene (50 mg per day, then a several days’ break, followed by 50 mg 2–3 times per day). After increasing dosages up to 250 mg per day, the patients had not experienced any improvements. Only 4 of them noticed a slight decrease of pain (2 patients with postherpetic neuralgia, 1 patient with thalamic syndrome and 1 patient with phantom pain after amputation). In the second study, a dosage of 50 mg every 6 h was used. With prolonged use, 7 of 22 patients reported a pain reduction by 75–100%, 9 patients did not notice any effect of the therapy, and the remaining 5 had a pain reduction by 50%. However, with long-term therapy and an increased dosage, some patients had side effects. The author concluded that in small doses, the drug was ineffective. With a higher dosage, a reduction in pain occurs in a third of patients, but the risk of serious side effects increases.

According to the results obtained in [256], patients with postherpetic pain showed a good response to chlorprothixene therapy and pain reduction at a dosage of 50 to 100 mg 4 times per day. Also, pain decrease was observed in patients with acute herpetic pain. However, in patients with post-laminectomy arachnoiditis, no improvement was observed. Summing up the study, the authors concluded that the drug was effective for herpetic and postherpetic neuralgia, but not very effective for pain associated with disorders of the central nerves of the spinal cord.

Since the last study was published in 1981, it seems that chlorprothixene as an analgesic drug has been forgotten. As for itching, analysis of the literature did not reveal any published work on the clinical trials of chlorprothixene as an antipruritic drug.

Our study showed the effectiveness of chlorprothixene for the treatment of chronic pruritus [22]. The study included 12 patients aged from 20 to 78 years: 4 men and 8 women with mood disorders and spontaneous diffuse itching, who were not satisfied by trazodone monotherapy (described in Section 3.5.2). For this group of patients, a new combination of therapy was tested based on the use of trazodone and chlorprothixene at a dosage of 15 mg per day. Before the start of chlorprothixene administration, the average intensity of itching in the group on the visual analogue scale (VAS) was 5.7 ± 2.0 points. The condition of the patients was checked within a week, and again after 4 weeks of therapy. In the first week of therapy, itching decreased to 4.5 ± 1.0 points on the VAS scale. A month later, the itching disappeared completely in all the studied patients, regardless of their age, sex and concomitant diseases. Thus, we proposed a universal antipruritic therapy for patients with diffuse itching associated with mood disorders and, hopefully, have given chlorprothixene a second chance.

In conclusion, chlorprothixene has shown its effectiveness in reducing chronic pain and itching in several clinical studies (Table 1). There were withdrawals and dropouts due to the following side effects: psychical abnormalities, drowsiness, and vertigo [254,255,256].

#### 3.6.3. Chlorprothixene. Interaction with the Receptors

**Figure 3:**
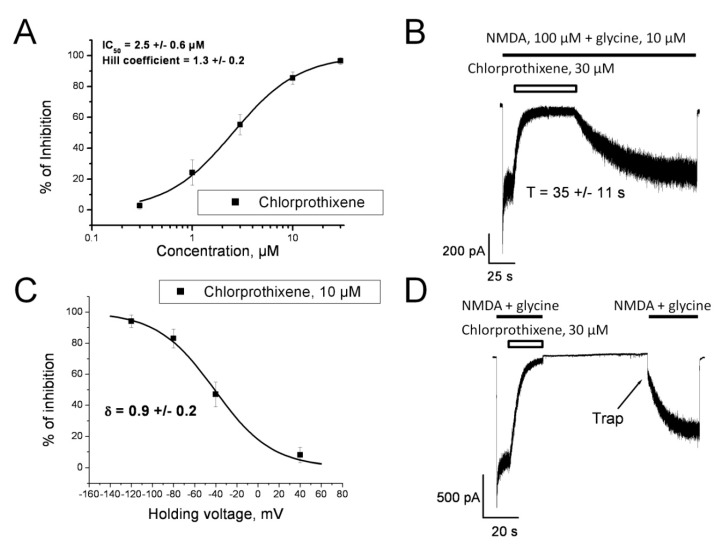
Action of chlorprothixene on NMDAR currents in isolated Wistar rat hippocampal CA1 pyramidal neurons. (**A**) Concentration dependence of action of chlorprothixene at −80 mV holding voltage in the absence of magnesium in extracellular solution was fitted by Hill equation. NMDAR currents were induced by 100 μM NMDA + 10 μM glycine. IC_50_ = 2.5 ± 0.6 µM and Hill coefficient = 1.3 ± 0.2. (**B**) Washout kinetics of chlorprothixene in the presence of agonists, weighted time constant from double-exponential fitting τ = 35 ± 11 s. (**C**) Voltage dependence of action of chlorprothixene fitted by Woodhull model. Δ = 0.9 ± 0.2, suggesting a binding site deep in the channel pore. (**D**) Chlorprothixene demonstrates partial trapping in the double-pulse protocol at −80 mV holding voltage.

Our own electrophysiological experiments showed that chlorprothixene was able to inhibit NMDAR, with IC_50_ = 2.5 ± 0.6 µM and a Hill coefficient equal to 1.3 ± 0.2 at −80 mV holding voltage in the absence of magnesium in extracellular solution (Figure 3A). It had rather slow washout kinetics; the weighted time constant T from for double-exponential fitting was 35 ± 11 s (Figure 3B). The inhibition by chlorprothixene was strongly voltage-dependent, zδ = 0.9 ± 0.2 according to Woodhull model (Figure 3C). Thus, its binding site is presumably located deep in the channel pore. In addition, inhibition by chlorprothixene was characterised by partial trapping (Figure 3D). All in all, its action on NMDAR resembled that of the structurally similar chlorpromazine, described by us previously [19].

Using the electrophysiological methods (5 Hz train protocol), a group of Hungarian scientists demonstrated that chlorprothixene can inhibit voltage-dependent sodium channels Nav1.2 in human HEK-293 cells with IC_50_ = 43 μM [198].

The papers [257,258] describe S-TBPS, which is the radioactive ligand that binds within the pore of the GABAAR channel (at picrotoxin binding site). GABA inhibits the binding of S-TBPS to the GABAAR, but some antipsychotics, including chlorprothixene, eliminate this inhibitory binding. Based on this result, the researchers concluded that these antipsychotics, including chlorprothixene, block GABAARs by binding within the channel pore. However, such an interaction cannot be associated with the antinociceptive effect of chlorprothixene, since the analgesic drugs acting through the GABAergic system are agonists but not blockers of this receptor.

The authors of [259] studied the interaction of antipsychotics, including chlorprothixene, with rat 5-HT7 receptors expressed in the COS-7 cell line. Atypical antipsychotics, including chlorprothixene, were shown to bind to the 5-HT7 receptors with high affinity. However, it is not discussed in the paper whether chlorprothixene acted as an agonist or antagonist. Since chlorprothixene is an antagonist of some other types of serotonin receptors [260], we assume that it was an antagonist. Therefore, this interaction cannot be associated with the antinociceptive effect of chlorprothixene, because the analgesic drugs that act through the serotonergic system are agonists, not blockers of these receptors.

It was shown in human histamine receptors expressed in the CHO cells that chlorprothixene is an effective antagonist of H1 receptors with an inhibition constant Ki = 4 nM [261].

There is indirect evidence that chlorprothixene can interact with TRP channels. Capsaicin is often used in experiments for potentiation of mechanical and thermal allodynia and hyperalgesia. Capsaicin binds to TRPV1 channels, activates them, sodium and calcium ions pass through the channels, and a nerve impulse is triggered. Resiniferatoxin is a capsaicin analogue with even greater activity against TRPV1 channels. Acs and co-authors studied the binding of radioactively labelled resiniferatoxin to TRPV1 channels and the effect of TCAs and phenothiazine-like antipsychotics on this binding [262]. Chlorprothixene was not studied, but chlorpromazine and cis-thiothixene, which are quite similar to it in structure and function, were. According to the data obtained, all studied drugs bind to TRPV1 channels at an allosteric site, different from the binding site of resiniferatoxin, and modulate the binding efficiency of the latter. Therefore, it is possible that chlorprothixene can also interact with the TRPV1 channel.

According to the literature, the effect of chlorprothixene on AMPA- receptors, calcium channels, opioid receptors, cannabinoid receptors, and the NCX has not been studied. The information on chlorprothixene pharmacological profile is summarised in Table 2.

### 3.7. Venlafaxine

#### 3.7.1. Venlafaxine. Animal Models

In the study [263] the antihyperalgesic effect of venlafaxine was tested in the CCI model in rats. Injured and control animals received a single (10, 20 and 40 mg/kg) or repeated (2.5, 5 and 10 mg/kg) subcutaneous injection of the drug. Using the hind paw pressure method, it was shown that venlafaxine has a dose-dependent effect. In a group of animals, who were injected repeatedly, the antinociceptive effect of venlafaxine was observed both in injured and healthy rats. To test whether the opioidergic system was involved in the antihyperalgesic effect of venlafaxine, naloxone was injected in mononeuropathic rats and did not inhibit the effect of continuous (5 × 10 mg) administration of the antidepressant. The antihyperalgesic effect was reversed by intraperitoneal administration of noradrenaline and serotonin synthesis inhibitors. These findings confirmed venlafaxine’s mechanism of action, but also showed the involvement of the monoaminergic system in chronic pain models.

The authors of [264] studied an antinociceptive effect of venlafaxine in rats with carrageenan-induced pain and inflammation. It was shown that it reduced mechanical hyperalgesia; a high dose of the drug was effective at each time point, and low doses were effective for up to 24 h. Moreover, venlafaxine was shown to have anti-inflammatory potential: 50 mg/kg and 100 mg/kg of the drug inhibited oedema formation, while 25 mg/kg did not. Similar results were obtained in [265]. The anti-inflammatory effect of venlafaxine in the rat model of carrageenan-induced paw oedema was studied. It was shown that intraperitoneal and intracerebroventricular administration of the drug inhibited paw oedema, reduced myeloperoxidase activity, and decreased interleukin (IL)-1β and tumour necrosis factor (TNF)-α production.

In [266], the dose-dependent (40, 60 and 80 mg/kg) reduction of mechanical hypersensitivity induced by SNI in adult rats was shown. The same results were observed with gabapentin treatment (60, 100 and 200 mg/kg). When these two drugs were combined and injected together, gabapentin improved the antinociceptive effect of the antidepressant in dosage 60 mg/kg. Such combined administration did not have any effect on gabapentin effect with the same dose. At the same time, the authors suggested that co-administration of these two drugs would have an additive-like effect, but further investigation needs to be done.

In the study [267], the antinociceptive effects of venlafaxine on heat hyperalgesia in the CCI model in rats was established. An interesting finding was that this effect was observed in both cases: (i) when the drug (10 and 20 mg/kg) was administrated daily for eleven days from the first day after injury, and (ii) when treatment (20 mg/kg) started at the eleventh day and continued until the twenty-first day after injury. Venlafaxine had no effect on cold and mechanical allodynia. Administration of yohimbine led to the attenuation of the antidepressant’s antinociceptive effect on heat hyperalgesia. The authors suggested involvement of α2-adrenergic receptors in the venlafaxine anti-pain effect.

Intriguing results were obtained by the authors of [268] in their experiments testing the effectiveness of venlafaxine in morphine tolerated mice. The drug (5 or 40 mg/kg) was injected prior to morphine administration, and a hot plate test was performed four days later. According to the data, venlafaxine prevented tolerance to high doses of morphine. Moreover, in these mice, the antidepressant inhibited the processes of oxidative stress and this effect was incremented by N(ω)-nitro-L-arginine methyl ester and aminoguanidine hydrochloride—the NO synthesis inhibitors. This data suggests that the drug attenuates neuroinflammation and oxidative stress in the brain of morphine-tolerant mice. The authors speculate that venlafaxine might be a supplemental drug to restore the morphine analgesic effect. Similar findings had been published earlier in [269], where the authors showed that venlafaxine (10 mg/kg) increased the anti-pain effect of morphine (5 mg/kg) in rats with induced diabetic neuropathy. However, chronic prolonged administration of the antidepressant decreases this effect of morphine. When rats were pretreated with naloxone, yohimbine (an α2-adrenoceptor antagonist), or p-chloroamphetamine (a neurotoxin that negatively affects serotoninergic neurons), an antinociceptive effect of venlafaxine and morphine administration was blocked.

#### 3.7.2. Venlafaxine. Clinical Studies

Rowbotham and co-authors [270] tested the efficacy of venlafaxine in a double-blind, placebo-controlled study in patients with diabetic neuropathic pain. 245 patients took part in this trial: 99 females and 145 males, with type I or type II diabetes, suffering from peripheral neuropathic pain. Patients were randomly divided into a group who received a placebo (n = 81), a group who received venlafaxine with a dose of 75 mg (n = 81) and a third group who took 150–225 mg of the drug (n = 82). The results of this 6 weeks study demonstrated a dose-dependent reduction of neuropathic pain in participants. It was suggested in earlier works that venlafaxine in lower doses affected serotonin reuptake, and when increasing the dose, it acted on norepinephrine reuptake as well. However, the question as to whether the analgesic effect of the drug was due to its action on norepinephrine or its balanced influence on both norepinephrine and serotoninergic systems still needs to be investigated. It has also been reported that side effects ranging from mild to moderate severity occurred during the treatment course. The effectiveness of venlafaxine was shown in [271] in a randomised, parallel-group, double-blind, double-dummy clinical trial in 66 patients with diabetic neuropathy.

Regarding inflammatory pain, Piletz and co-authors reported concave significant associations between pro-inflammatory biomarkers (tumour necrosis factor (TNF)-α, interleukin (IL)-1β, and monocyte chemotactic protein-1) and plasma levels of venlafaxine in patients with a major depressive disorder [272].

Lee et al. in 2010 investigated the effect of venlafaxine with a dosage of 75 mg/day in males with functional non-cardiac chest pain [273]. The study was a two-treatment, two-period, two-sequence crossover trial. After 4 weeks of treatment, more than a half of participants had improved pain scores without serious side effects. The authors suggested that the drug decreased visceral hyperalgesia by effecting serotoninergic transmission. Despite the positive results, this study had limitations: the low number (n = 43) and young age of participants.

A comparative, double-blind clinical trial with venlafaxine, carbamazepine, and pregabalin was made by Razazian and co-authors [274]. The patients (females, n = 257) suffering from painful diabetic neuropathy were randomly divided into three groups according to the prescribed drug, while the placebo group wasn’t included. During treatment, the dosage of each drug increased after the first week: carbamazepine from 100 mg to 200 mg every 12 h; venlafaxine from 75 mg to 150 mg daily; pregabalin from 75 mg daily up to 75 mg every twelve hours. Interestingly, patients were also allowed to take acetaminophen not more than 4 g/day. According to the results, all three drugs were effective in pain reduction: pregabalin and venlafaxine in 75 mg twice a day and carbamazepine in 200 mg twice a day, but pregabalin was the most effective. At the same time, in this group, reports of side effects such as dizziness and somnolence were more frequent than in groups with venlafaxine and carbamazepine.

The authors of both [271,274] noted the maximum decrease in pain in the second week of venlafaxine treatment, while in [274], the dosage was three times lower—25 mg/twice a day. This diversity may be due to different populations taking a part in trials.

A comprehensive Cochrane review of venlafaxine efficacy in neuropathic pain was published by Gallagher and colleagues in 2015 [275]. The authors analysed the results of the drug pain treatment of six randomised, double-blind trials. They pointed out that this work’s results were unconvincing due to methodological limitations, and the side effects of venlafaxine reported in some reviewed studies. They concluded that the drug had no particular benefits over existing treatments for neuropathic pain.

The authors of [276] tested the effects of venlafaxine and duloxetine on chemotherapy-induced peripheral neuropathy in a double-blind clinical trial. The patients (n = 152) with cancer that were receiving and had developed chemotherapy-induced peripheral neuropathy were randomly divided into three pharmacotherapy groups, treated with venlafaxine, duloxetine and placebo, respectively, with 52 patients in each group. The doses of venlafaxine (capsules of 37.5 mg) and duloxetine (capsules of 30 mg) were determined once daily. The study continued for four weeks. In the venlafaxine and duloxetine groups, but not in the placebo group, cranial neuropathy grade, sensory neuropathy and neuropathic pain grade decreased significantly by the end of the study period. However, this reduction was more considerable in the duloxetine group compared to venlafaxine group. The authors concluded that because of the high cost of duloxetine, venlafaxine could be considered as the first-line medicine in the management of chemotherapy-induced peripheral neuropathy, and in case of no response, duloxetine can be used.

Thus, in several clinical studies, venlafaxine has shown its effectiveness in reducing chronic pain (predominantly in diabetic neuropathy). As for itching, we could not find any published trials on the antipruritic effect of venlafaxine. The following adverse effects of the drug were reported: nausea, somnolence, headache, insomnia, sexual dysfunction, and dizziness [270,273,274,276].

#### 3.7.3. Venlafaxine. Interaction with the Receptors

With the help of extrinsic fluorescence quenching, it was demonstrated that venlafaxine is able to bind to the extracellular S1S2 domain of the NR1-1b subunit of the NMDA receptor, but not in clinically relevant concentrations [277]. It was shown that venlafaxine takes part in maintaining NMDA receptors, and upregulating Grin2a and Grin2b expression [278,279]. Venlafaxine treatment upregulated GRIA3 gene (glutamate receptor AMPA 3) [279]. These findings point to the involvement of the glutamatergic system in the antidepressant effect of the drug, and the analgesic effect of venlafaxine is probably not connected with anti-glutamate activity.

Using the patch-clamp technique, the authors of [280] showed in isolated guinea pigs’ ventricular myocytes that venlafaxine inhibits peak I(Na) in a concentration-dependent manner, with an estimated IC_50_ of 8 μM. Moreover, according to these results, venlafaxine binds to the resting state of the channel, while most tricyclic antidepressants bind to the inactivated state.

In the paper [281], the antinociceptive effect of venlafaxine was measured in mice by the hot plate test. Venlafaxine induced a dose-dependent antinociceptive effect, which was abolished by naloxone, norbinaltorphimine (κ-opioid receptor antagonist) and naltrindole (δ-opioid receptor antagonist), but not by beta-funaltrexamine (μ-opioid receptor antagonist). The authors suggested the involvement of κ- and δ-opioid mechanisms in an analgesic action of venlafaxine. A similar result was described in [282]: the antinociceptive effect of venlafaxine measured in mice by the tail flick test was antagonised by naloxone. The authors of [263] obtained the opposite data. Venlafaxine was shown to have the anti-hyperalgesic action in diabetic rats with the paw pressure test. This effect was not reversed by naloxone, which means that it does not involve the endogenous opioid system.

According to [283], venlafaxine was not able to bind with 5-HT7 receptors in clinically relevant concentrations. Venlafaxine is reported to have no obvious affinity towards histamine receptors [284,285].

The effect of venlafaxine on TRP channels has not been studied yet. However, the authors of [286] showed with the help of the patch-clamp technique that tramadol (another SNRI, whose structure is similar to venlafaxine) selectively inhibits the function of TRPA1, but not that of TRPV1 receptors, and that TRPA1 may play a role in the analgesic effects of the drug. Therefore, it may be possible that venlafaxine is also able to affect TRP channels.

We did not find any published data on the effect of venlafaxine on calcium channels, GABAAR, the NCX or cannabinoid receptors. The information regarding the venlafaxine pharmacological profile is summarised in Table 2.

## 4. Discussion

Having compared the data on the analgesic/antipruritic efficacy of drugs (Table 1) and their pharmacological profile (Table 2), we have made some observations and gathered them in Table 5.

The voltage-dependent sodium channels involved in nociception (Nav1.7, Nav1.8 and Nav1.9) are predominantly expressed in the PNS: in the dorsal root ganglion (DRG) and the trigeminal ganglion [287]. Their role in PNS disorders has been proved [288,289]. Therefore, as expected, the drugs that are able to block these types of sodium channels are effective in the management of pain caused by peripheral nerve injury (Table 5). Therefore, trazodone, chlorprothixene, carbamazepine, venlafaxine are effective in diabetic neuropathy and postherpetic neuralgia in humans, and mianserin is effective in diabetic neuropathy in animal models. At the same time, these drugs are able to reduce the ion current through voltage-dependent sodium channels. Citalopram, on the one hand, is a weak inhibitor of Nav1.8 (IC_50_ = 100 μM). On the other hand, it provides slight pain relief in diabetic neuropathy. We don’t believe that it can be excluded that modulation of sodium current by citalopram contributes to its antinociceptive effect in diabetic neuropathy.

As was expected, the drugs modulating opioid and cannabinoid receptors have been proven to be effective as supraspinal analgesia (Table 5). Several clinical studies and animal experiments have shown that citalopram affects the emotional component of pain, and, according to the literature, it interacts with the cannabinoid system. Mianserin, trazodone and venlafaxine, on the one hand, have shown an analgesic effect in the hot plate test (when the jumps of an animal indicate the involvement of the supraspinal component of pain behaviour). On the other hand, the effect of these drugs on opioid receptors has been proven.

Analysis of the information collected leads us to suggest that the interaction of mianserin with the κ-opioid receptors makes a considerable contribution to the analgesic effect. According to the data reported in the literature, the distribution, function and pharmacology of these receptors depend on sex [290,291], and our clinical trials have shown that mianserin (as an analgesic drug) is much more effective for men than for women (Table 3 and Table 5). Tianeptine interacts with the μ-opioid receptors, and but not with the κ-opioid receptors. It was shown in animal models that sex has little effect on the function of the μ-opioid receptors and the age of the animals has a much stronger effect [292]. According to our experiments, the analgesic efficacy of tianeptine is the same for men and women (Table 3 and Table 5). Maybe tianeptine would have a different effect on young and elderly patients, but we did not check this possibility.

In connection with these observations, it is important to mention that a large amount of evidence has been accumulated so far indicating that pain pathways are different for men and for women, and sex should be a key factor in personalised prescriptions [293].

It is interesting that almost all compounds (except carbamazepine, tianeptine and venlafaxine) are inhibitors of histamine receptors, despite their different structures. The interaction of carbamazepine with histamine receptors has not been studied, so it is not excluded. Perhaps the interaction of these drugs with histamine receptors explains their antipruritic effect.

However, there are contradictions in this assumption. The anxiolytic effect of the histamine H1 receptor antagonist pyrilamine was studied in animal models [294]. It was found that female mice were more susceptible to the drug than males. This is not consistent with our clinical experiments, which showed that carbamazepine and mianserin were more effective for treating pruritus in men than in women. Therefore, it seems that the antipruritic effect of the studied drugs is more complex than the simple inhibition of H1 receptors (Table 5). Tianeptine is not a histamine receptor inhibitor, and it is a more effective antipruritus drug for men too. Its antipruritic effect is apparently due to interaction with some other targets, possibly with TRP channels. Antipsychotic chlorpromazine and cis-thiothixene, which are quite similar to chlorprothixene in structure and function, bind to TRPV1 channels. Therefore, it is quite possible that chlorprothixene can also interact with TRPV1 channel. On the other hand, the drug has antihistamine activity (Table 1), which means that this compound, according to our speculation, can hit two antipruritic targets. As mentioned in Section 3.6.2, our clinical studies showed 100% antipruritic effectiveness of low doses of chlorprothixene in combination with trazodone in patients suffering with diffuse itching [22] (Table 5).

Experiments with animal models have shown that tianeptine, citalopram, carbamazepine, trazodone and venlafaxine are effective against inflammatory pain caused by formalin, carrageenan and brewer’s yeast (Table 1 and Table 5). Proinflammatory cytokines are involved in the development of formalin-induced inflammatory pain in rodents [295,296]. On the other hand, many studies have shown that depressed patients have increased production of proinflammatory cytokines [297,298,299]. Therefore, it cannot be discounted that the anti-inflammatory effect contributes the antidepressant effect of tianeptine, citalopram and trazodone. Carbamazepine is not an antidepressant, but had a slight antidepressant effect on our patients with CRF and chronic pain (Table 4). Its antidepressant effect was shown in [300], as well.

Our analysis has revealed a huge number of “blank spots” in the mechanisms of the analgesic and, especially, the antipruritic effects of psychotropic drugs. The interaction of psychotropics with TRP channels, which are key players in transmitting a pruriceptive signal, has not been investigated adequately. The antipruritic effect of the drugs has not been studied in animal models at all. Also, we have found no explanation for the fact that, in our clinical studies, mianserin, carbamazepine and tianeptine suppress neuropathic itching in men more effectively than in women (Table 3). Perhaps this is somehow connected with the emotional component of itching. Or it is possible that these drugs affect TRP channels, and TRP channels in turn have different distribution or/and functions in male and female organisms.

The mechanisms of neuropathic pain and the analgesic effect of psychotropic drugs have been studied much better than pruritus. However, there are still many questions; most of all, in our opinion, regarding the role of calcium ions. 

First of all, there is no clear data for some drugs with respect to their ability to inhibit calcium channels. Secondly, the contribution of the sodium-calcium exchanger to the pharmacological profile of psychotropic drugs has not been studied at all. Thirdly, our review describing just seven compounds could not reveal any certain relationships between the type of their analgesic activity and their ability to modulate calcium flux through the calcium channels. This question requires further investigation.

Having analysed the clinical data of the analgesic and antipruritic efficacy of these seven drugs, we found that almost no-one divides patients into groups by sex and/or age, although such studies are important for effective therapy development. We were practically the only ones who tried to analyse the efficacy of drugs separately in men and women, and such studies on large groups of patients are desirable.

## 5. Conclusions

Chronic pain/itching and mood disorders accompany each other frequently. Much information has been obtained so far indicating that the molecular mechanisms of these pathologies overlap. Enhancement and ordering of our knowledge of the analgesic/antipruritic action of psychotropic drugs is an actual problem. The information will help to develop the personalised medicine and reduce the number of prescribed drugs and side effects. There is no doubt that work on this topic requires the continuation.

## Figures and Tables

**Table 1:** Analgesic and antipruritic effect of psychotropic drugs on different types of pain/itching.

Drug	Analgesic Effect: Animal Models	Analgesic Effect: Clinical Trial	Antipruritic Effect: Animal Models	Antipruritic Effect: Clinical Trial	Dosage in Clinical Trials, Literature Data (Our Study)	Main Side Effects Led to Withdrawals and Refusal of Treatment
Tianeptine	formalin test; mechanical allodynia; fibromyalgia	chronic pain in patients with chronic renal failure (CRF)	ND	neuropathic pruritus in male patients with CRF	20–40 mg/day (37.5 mg/day)	ND *
Citalopram	hot plate test; the second phase of the formalin test; thermal hypersensitivity in the chronic constriction injury (CCI) model	psychosomatic pain; diabetic neuropathy (mild effect); migraine (presumably); neuropathic pain in patients with CRF (mild effect)	ND	itching in psoriasis (mild effect); neuropathic pruritus in patients with CRF (mild effect)	20–40 mg/day (20 mg/day)	nausea, vomiting, epileptic seizures, tachycardia, dizziness, gastric upset, diarrhea
Mianserin	hot plate test (enhancement of metamizol); tail flick test (enhancement of metamizole and indometacin); diabetic neuropathy	neuropathic pain in patients with CRF; tension headache; chronic pain associated with “true” depression	potentiation of hindlimb scratching	neuropathic pruritus in male patients with CRF	30–90 mg/day (30 mg/day)	lack of appetite, dryness of mouth, thirst, drowsiness, dullness
Carbamazepine	hot plate test; inflammatory pain; trigeminal neuralgia; chemotherapy-induced painful neuropathy	diabetic neuropathy; trigeminal neuralgia	ND	itching in sclerosis; brachioodal itch; trigeminal trophic syndrome	200–800 mg/day (300 mg/day)	giddiness, rash, sleeplessness
Trazodone	hot plate test; thermal hyperalgesia; formalin induced pain	diabetic neuropathy; adjuvant therapy in fibromyalgia	ND	diffuse pruritus	50–300 mg/day (50–100 mg/day)	dizziness, headache, tachycardia, stomachache
Chlorprothixene	anesthesia of experimental animals	postherpetic neuralgia; adjuvant therapy in meralgia paraesthetica	ND	diffuse pruritus	50–400 mg/day (15 mg/day)	psychical abnormalities, drowsiness, vertigo
Venlafaxine	hyperalgesia in the CCI model; carrageenan-induced pain and inflammation; mechanical hypersensitivity induced by SNI; neuroinflammation and oxidative stress	diabetic neuropathy; chemotherapy-induced peripheral neuropathy; visceral hyperalgesia; inflammation	ND	ND	37.5–225 mg/day	nausea, somnolence, headache, insomnia, sexual dysfunction, dizziness

“ND”—no data, *—there are no data about side effects of tianeptine in experiments on its analgesic/antipruritic efficacy, but the drug has neurologic, cardiovascular, and gastrointestinal side effects as an antidepressant.

**Table 2:** Pharmacological profile of psychotropic drugs.

Drug	NMDA (inhib.)	AMPA (inhib.)	Sodium (inhib.)	Calcium (inhib.)	GABA(A) (activ.)	Opioid (activ.)	Cannabinoid (modul.)	5HT7 (activ.)	NCX (inhib.)	Histamine (inhib.)	TRP (inhib.)
Tianeptine	−	−	ND	−	−	+	ND	+	ND	−	ND
Citalopram	−	−	weak	+	−	−	+	−	ND	+	ND
Mianserin	−	−	+	there are + and −	−	+	ND	−	ND	+	ND
Carbamazepine	−	−	+	there are + and −	+	ND	possible	ND	ND	ND	−
Trazodone	−	ND	there are + and −	+	+	+	ND	+	ND	+	ND
Chlorprothixene	+	ND	+	ND	−	ND	ND	−	ND	+	possible
Venlafaxine	−	ND	+	ND	ND	+	ND	−	ND	−	possible

“inhib.”—inhibition, “activ.”—activation, “modul.”—modulation, “+”—interaction has been shown, “−“—lack of interaction has been shown, “ND”—no data, “possible”—interaction has been shown for similar drugs.

**Table 3:** Treatment of chronic pain and itching of patients with chronic renal failure in chronic haemodialysis with antidepressants) [9].

**Pain (Dartmouth Pain Questionnaire)**				
**Drug**	**Men**		**Women**	
	**Before Treatment**	**After Treatment**	**Before Treatment**	**After Treatment**
tianeptine	0.44 ± 0.21	0.21 ± 0.16	0.91 ± 0.16	0.45 ± 0.21
citalopram	0.78 ± 0.25	0.64 ± 0.18	0.85 ± 0.16	0.66 ± 0.12
mianserin	0.81 ± 0.23	0.27 ± 0.06	0.93 ± 0.18	0.71 ± 0.16
comparison group	0.13 ± 0.07	0.13 ± 0.1	0.23 ± 0.12	0.22 ± 0.11
**Itch, Scoring Atopic Dermatitis index scale (SCORAD)**				
**Drug**	**Men**		**Women**	
	**Before Treatment**	**After Treatment**	**Before Treatment**	**After Treatment**
tianeptine	17.4 ± 8.1	10.8 ± 5.6	15.5 ± 1.3	12.5 ± 1.7
citalopram	16.45 ± 6.7	14.45 ± 6.0	13.75 ± 1.8	10.75 ± 1.3
mianserin	18 ± 7.5	4.75 ± 1.8	8.41 ± 2.1	6.97 ± 1.3
comparison group	9.16 ± 3.44	9.42 ± 3.6	7.64 ± 1.8	7.96 ± 1.6

**Table 4:** Reduction of pain and itching syndromes, depression and anxiety in the process of carbamazepine treatment in eleven patients with chronic renal failure in chronic haemodialysis [21].

Patients	Pain	Itch	Depression	Anxiety
men	76.1%	84.0%	20.9%	31.1%
women	63.1%	44.0%	18.9%	30.0%

**Table 5:** Proposed relationships between pharmacological profiles and analgesic/antipruritic effect of the psychotropic drugs studied.

Drugs	Receptors	Type of Analgesic/Antipruritic Effect (Animal Models or/and Clinical Studies)
Trazodone chlorprothixene carbamazepine mianserin	voltage-dependent sodium channels in the peripheral nervous system	diabetic neuropathy, postherpetic neuralgia
citalopram mianserin trazodone venlafaxine	cannabinoid or opioid receptors	supraspinal analgesia
mianserin	κ-opioid receptors	sex-dependent analgesia
tianeptine	μ-opioid receptors	sex-independent analgesia
tianeptine citalopram trazodone venlafaxine carbamazepine	no similarities in pharmacological profiles found	inflammatory pain/antidepressants (or has antidepressant action)
chlorprothixene	histamine receptors + possible TRP channel *	sex-independent antipruritic action
mianserin carbamazepine tianeptine	no similarities in pharmacological profiles found	sex-dependent antipruritic action

* interaction with TRP channel was shown for chlorpromazine and cis-thiothixene, which are similar to chlorprothixene in structure and function.

## References

[1] Murtagh F.E., Addington-Hall J., Higginson I.J. (2007). The prevalence of symptoms in end-stage renal disease: a systematic review. Adv. Chronic Kidney Dis..

[2] Schreiber A.K., Nones C.F., Reis R.C., Chichorro J.G., Cunha J.M. (2015). Diabetic neuropathic pain: Physiopathology and treatment. World J. Diabetes.

[3] Fallon M.T. (2013). Neuropathic pain in cancer. Br. J. Anaesth..

[4] Andrew D., Schmelz M., Ballantyne J.C., Dostrovsky J.O., Carr D.B., Koltzenburg M. (2003). Itch: Mechanisms and mediators. Progress in Pain Research and Management.

[5] Carstensa E., Akiyama T. (2016). Central mechanisms of itch. Curr. Probl. Dermatol..

[6] Ferm I., Sterner M., Wallengren J. (2010). Somatic and psychiatric comorbidity in patients with chronic pruritus. Acta Derm. Venereol..

[7] Torta R., Ieraci V., Zizzi F. (2017). A Review of the Emotional Aspects of Neuropathic Pain: From Comorbidity to Co-Pathogenesis. Pain Ther..

[8] Bair M.J., Robinson R.L., Katon W., Kroenke K. (2003). Depression and pain comorbidity: A literature review. Arch. Intern. Med..

[9] Vanchakova N.P., Rybakova K.V., Smirnov A.V., Shestakova N.N. (2003). Specific use of antidepressants of different chemical groups in patients with chronic renal failure and syndromes of itching and pain receiving chronic hemodialysis. Nephrology (Saint-Petersburg).

[10] Mika J., Zychowska M., Makuch W., Rojewska E., Przewlocka B. (2013). Neuronal and immunological basis of action of antidepressants in chronic pain—Clinical and experimental studies. Pharmacol. Rep..

[11] Yosipovitch G., Bernhard J.D. (2013). Clinical practice. Chronic pruritus. N. Engl. J. Med..

[12] Baltenberger E.P., Buterbaugh W.M., Martin B.S., Thomas C.J. (2015). Review of antidepressants in the treatment of neuropathic pain. Ment. Health Clin..

[13] Jensen T.S. (2002). Anticonvulsants in neuropathic pain: Rationale and clinical evidence. Eur. J. Pain.

[14] Seidel S., Aigner M., Ossege M., Pernicka E., Wildner B., Sycha T. (2010). Antipsychotics for acute and chronic pain in adults. J. Pain Symptom Manag..

[15] Shestakova N.N., Vanchakova N.P. (2006). Theoretical conformational analysis of antidepressant as a way for evaluation of their efficiency for pain and itch syndrome management in patients with end-stage renal disease under chronic hemodialysis. Dokl. Biochem. Biophys..

[16] Sernagor E., Kuhn D., Vyklicky L., Mayer M.L. (1989). Open channel block of NMDA receptor responses evoked by tricyclic antidepressants. Neuron.

[17] Watanabe Y., Saito H., Abe K. (1993). Tricyclic antidepressants block NMDA receptor-mediated synaptic responses and induction of long-term potentiation in rat hippocampal slices. Neuropharmacology.

[18] Barygin O.I., Gmiro V.E., Kim K.K., Magazanik L.G., Tikhonov D.B. (2009). Blockade of NMDA receptor channels by 9-aminoacridine and its derivatives. Neurosci. Lett..

[19] Barygin O.I., Nagaeva E.I., Tikhonov D.B., Belinskaya D.A., Vanchakova N.P., Shestakova N.N. (2017). Inhibition of the NMDA and AMPA receptor channels by antidepressants and antipsychotics. Brain Res..

[20] Vanchakova N.P., Smirnov A.V., Rybakova K.V., Khalezova N.B., Shestakova N.N. (2006). Comparative clinical studies of mianserin and carbamazepine efficacy for pain and itch management in patients with end-renal disease under chronic hemodialysis. Eur. J. Pain.

[21] Shestakova N.N., Vanchakova N.P. (2008). The technology of detection among antidepressants and anticonvulsants the medicines for management of itch and pain syndromes using computer modeling methods. Alm. Clin. Med..

[22] Shestakova N.N., Belinskaya D.A., Barygin O.I., Vanchakova N.P. The complex therapy for patients suffered from diffuse pruritus. Proceedings of the 16th World Congress on Pain.

[23] Barygin O.I. (2019). Personal communication.

[24] Belinskaia D.A., Goncharov N.V., Shestakova N.N., Zinchenko V.P., Berezhnov A.V. (2017). Mechanism of the Analgesic Action of Ppsychotropic Drugs: Interaction with the Transport Protein Albumin and NMDA-Receptors. Proceedings of the International Conference on Receptors and Intracellular Signaling.

[25] Gillman P.K. (2007). Tricyclic antidepressant pharmacology and therapeutic drug interactions updated. Br. J. Pharmacol..

[26] Kremer M., Salvat E., Muller A., Yalcin I., Barrot M. (2016). Antidepressants and gabapentinoids in neuropathic pain: Mechanistic insights. Neuroscience.

[27] Fornasari D. (2017). Pharmacotherapy for Neuropathic Pain: A Review. Pain Ther..

[28] Chincholkar M. (2018). Analgesic mechanisms of gabapentinoids and effects in experimental pain models: A narrative review. Br. J. Anaesth..

[29] Ishida J.H., McCulloch C.E., Steinman M.A., Grimes B.A., Johansen K.L. (2018). Gabapentin and Pregabalin Use and Association with Adverse Outcomes among Hemodialysis Patients. J. Am. Soc. Nephrol..

[30] Bleakman D., Alt A., Nisenbaum E.S. (2006). Glutamate receptors and pain. Semin. Cell Dev. Biol..

[31] Gangadharan V., Wang R., Ulzhöfer B., Luo C., Bardoni R., Bali K.K., Agarwal N., Tegeder I., Hildebrandt U., Nagy G.G. (2011). Peripheral calcium-permeable AMPA receptors regulate chronic inflammatory pain in mice. J. Clin. Investig..

[32] Medvedev I.O., Malyshkin A.A., Belozertseva I.V., Sukhotina I.A., Sevostianova N.Y., Aliev K., Zvartau E.E., Parsons C.G., Danysz W., Bespalov A.Y. (2004). Effects of low-affinity NMDA receptor channel blockers in two rat models of chronic pain. Neuropharmacology.

[33] Maier C., Dertwinkel R., Mansourian N., Hosbach I., Schwenkreis P., Senne I., Skipka G., Zenz M., Tegenthoff M. (2003). Efficacy of the NMDA-receptor antagonist memantine in patients with chronic phantom limb pain—Results of a randomized double-blinded, placebo-controlled trial. Pain.

[34] Hudspith M.J., Harrisson S., Smith G., Bountra C., Elliot P.J., Birch P.J., Hunt S.P., Munglani R. (1999). Effect of post-injury NMDA antagonist treatment on long-term Fos expression and hyperalgesia in a model of chronic neuropathic pain. Brain Res..

[35] Bilsky E.J., Inturrisi C.E., Sadée W., Hruby V.J., Porreca F. (1996). Competitive and non-competitive NMDA antagonists block the development of antinociceptive tolerance to morphine, but not to selective mu or delta opioid agonists in mice. Pain.

[36] Christoph T., Reissmüller E., Schiene K., Englberger W., Chizh B.A. (2005). Antiallodynic effects of NMDA glycine(B) antagonists in neuropathic pain: possible peripheral mechanisms. Brain Res..

[37] Coderre T.J., Van Empel I. (1994). The utility of excitatory amino acid (EAA) antagonists as analgesic agents. I. Comparison of the antinociceptive activity of various classes of EAA antagonists in mechanical, thermal and chemical nociceptive tests. Pain.

[38] Bereiter D.A., Bereiter D.F., Hathaway C.B. (1996). The NMDA receptor antagonist MK-801 reduces Fos-like immunoreactivity in central trigeminal neurons and blocks select endocrine and autonomic responses to corneal stimulation in the rat. Pain.

[39] Pappagallo M. (2005). Neurological Basis of Pain.

[40] Kristensen J.D., Svensson B., Gordh T. (1992). The NMDA-receptor antagonist CPP abolishes neurogenic ‘wind-up pain’ after intrathecal administration in humans. Pain.

[41] Wallace M., White A., Grako K.A., Lane R., Cato A.J., Snodgrass H.R. (2017). Randomized, double-blind, placebo-controlled, dose-escalation study: Investigation of the safety, pharmacokinetics, and antihyperalgesic activity of l-4-chlorokynurenine in healthy volunteers. Scand. J. Pain.

[42] Meymandi M.S., Keyhanfar F., Sepehri G.R., Heravi G., Yazdanpanah O. (2017). The Contribution of NMDA Receptors in Antinociceptive Effect of Pregabalin: Comparison of Two Models of Pain Assessment. Anesth. Pain Med..

[43] Kato E., Matsuzawa R., Kobayashi S., Fukushima T., Maekawa M., Hori Y. (2017). Effects of pregabalin on spinal d-serine content and NMDA receptor-mediated synaptic transmission in mice with neuropathic pain. Neurosci. Lett..

[44] Pieri C., Recchioni R., Moroni F., Balkay L., Márián T., Trón L., Damjanovich S. (1989). Ligand and voltage gated sodium channels may regulate electrogenic pump activity in human, mouse and rat lymphocytes. Biochem. Biophys. Res. Commun..

[45] Mantegazza M., Catterall W.A., Noebels J.L., Avoli M., Rogawski M.A., Olsen R.W., Delgado-Escueta A.V. (2012). Voltage-Gated Na+ Channels: Structure, Function, and Pathophysiology. Jasper’s Basic Mechanisms of the Epilepsies.

[46] Goldin A.L., Barchi R.L., Caldwell J.H., Hofmann F., Howe J.R., Hunter J.C., Kallen R.G., Mandel G., Meisler M.H., Netter Y.B. (2000). Nomenclature of voltage-gated sodium channels. Neuron.

[47] Devor M. (2006). Sodium channels and mechanisms of neuropathic pain. J. Pain.

[48] Dick I.E., Brochu R.M., Purohit Y., Kaczorowski G.J., Martin W.J., Priest B.T. (2007). Sodium channel blockade may contribute to the analgesic efficacy of antidepressants. J. Pain.

[49] Fozzard H.A., Sheets M.F., Hanck D.A. (2011). The sodium channel as a target for local anesthetic drugs. Front. Pharmacol..

[50] Maatuf Y., Geron M., Priel A. (2019). The Role of Toxins in the Pursuit for Novel Analgesics. Toxins.

[51] Cardoso F.C., Lewis R.J. (2019). Structure-Function and Therapeutic Potential of Spider Venom-Derived Cysteine Knot Peptides Targeting Sodium Channels. Front. Pharmacol..

[52] Cox J.J., Reimann F., Nicholas A.K., Thornton G., Roberts E., Springell K., Karbani G., Jafri H., Mannan J., Raashid Y. (2006). An SCN9A channelopathy causes congenital inability to experience pain. Nature.

[53] Yang Y., Mis M.A., Estacion M., Dib-Hajj S.D., Waxman S.G. (2018). NaV1.7 as a Pharmacogenomic Target for Pain: Moving Toward Precision Medicine. Trends Pharmacol. Sci..

[54] Wu Y.J., Guernon J., Shi J., Ditta J., Robbins K.J., Rajamani R., Easton A., Newton A., Bourin C., Mosure K. (2017). Development of New Benzenesulfonamides As Potent and Selective Nav1.7 Inhibitors for the Treatment of Pain. J. Med. Chem..

[55] Kornecook T.J., Yin R., Altmann S., Be X., Berry V., Ilch C.P., Jarosh M., Johnson D., Lee J.H., Lehto S.G. (2017). Pharmacologic Characterization of AMG8379, a Potent and Selective Small Molecule Sulfonamide Antagonist of the Voltage-Gated Sodium Channel NaV1.7. J. Pharmacol. Exp. Ther..

[56] Zeng X., Li P., Chen B., Huang J., Lai R., Liu J., Rong M. (2018). Selective Closed-State Nav1.7 Blocker JZTX-34 Exhibits Analgesic Effects against Pain. Toxins.

[57] Tikhonov D.B., Zhorov B.S. (2017). Mechanism of sodium channel block by local anesthetics, antiarrhythmics, and anticonvulsants. J. Gen. Physiol..

[58] Buyan A., Sun D., Corry B. (2018). Protonation state of inhibitors determines interaction sites within voltage-gated sodium channels. Proc. Natl. Acad. Sci. USA.

[59] Striggow F., Ehrlich B.E. (1996). Ligand-gated calcium channels inside and out. Curr. Opin. Cell Biol..

[60] Catterall W.A. (2000). Structure and regulation of voltage-gated Ca2+ channels. Annu. Rev. Cell Dev. Biol..

[61] Ertel E.A., Campbell K.P., Harpold M.M., Hofmann F., Mori Y., Perez-Reyes E., Schwartz A., Snutch T.P., Tanabe T., Birnbaumer L. (2000). Nomenclature of voltage-gated calcium channels. Neuron.

[62] Davies J.N., Zamponi G.W. (2008). Old proteins, developing roles: The regulation of calcium channels by synaptic proteins. Channels (Austin).

[63] Zamponi G.W., Lewis R.J., Todorovic S.M., Arneric S.P., Snutch T.P. (2009). Role of voltage-gated calcium channels in ascending pain pathways. Brain Res. Rev..

[64] Catterall W.A., Dib-Hajj S., Meisler M.H., Pietrobon D. (2008). Inherited neuronal ion channelopathies: New windows on complex neurological diseases. J. Neurosci..

[65] Schroeder C.I., Doering C.J., Zamponi G.W., Lewis R.J. (2006). N-type calcium channel blockers: Novel therapeutics for the treatment of pain. Med. Chem..

[66] Gohil K., Bell J.R., Ramachandran J., Miljanich G.P. (1994). Neuroanatomical distribution of receptors for a novel voltage-sensitive calcium-channel antagonist, SNX-230 (omega-conopeptide MVIIC). Brain Res..

[67] Catterall W.A., Few A.P. (2008). Calcium channel regulation and presynaptic plasticity. Neuron.

[68] Chaplan S.R., Pogrel J.W., Yaksh T.L. (1994). Role of voltage-dependent calcium channel subtypes in experimental tactile allodynia. J. Pharmacol. Exp. Ther..

[69] Matthews E.A., Dickenson A.H. (2001). Effects of spinally delivered N- and P-type voltage-dependent calcium channel antagonists on dorsal horn neuronal responses in a rat model of neuropathy. Pain.

[70] Evans A.R., Nicol G.D., Vasko M.R. (1996). Differential regulation of evoked peptide release by voltage-sensitive calcium channels in rat sensory neurons. Brain Res..

[71] McGivern J.G., McDonough S.I. (2004). Voltage-gated calcium channels as targets for the treatment of chronic pain. Curr. Drug Targets CNS Neurol. Disord..

[72] Perret D., Luo Z.D. (2009). Targeting voltage-gated calcium channels for neuropathic pain management. Neurotherapeutics.

[73] Chen J., Li L., Chen S.R., Chen H., Xie J.D., Sirrieh R.E., MacLean D.M., Zhang Y., Zhou M.H., Jayaraman V. (2018). The α2δ-1-NMDA Receptor Complex is Critically Involved in Neuropathic Pain Development and Gabapentin Therapeutic Actions. Cell Rep..

[74] Todorovic S.M., Jevtovic-Todorovic V. (2006). The role of T-type calcium channels in peripheral and central pain processing. CNS Neurol. Disord. Drug Targets.

[75] Sekizawa S.I., French A.S., Torkkeli P.H. (2000). Low-voltage-activated calcium current does not regulate the firing behavior in paired mechanosensory neurons with different adaptation properties. J. Neurophysiol..

[76] M’Dahoma S., Gadotti V.M., Zhang F.X., Park B., Nam J.H., Onnis V., Balboni G., Lee J.Y., Zamponi G.W. (2016). Effect of the T-type channel blocker KYS-05090S in mouse models of acute and neuropathic pain. Pflugers Arch..

[77] Pudukulatham Z., Zhang F.X., Gadotti V.M., M’Dahoma S., Swami P., Tamboli Y., Zamponi G.W. (2016). Synthesis and characterization of a disubstituted piperazine derivative with T-type channel blocking action and analgesic properties. Mol. Pain.

[78] El Alaoui C., Chemin J., Fechtali T., Lory P. (2017). Modulation of T-type Ca2+ channels by Lavender and Rosemary extracts. PLoS ONE.

[79] Kerckhove N., Pereira B., Soriot-Thomas S., Alchaar H., Deleens R., Hieng V.S., Serra E., Lanteri-Minet M., Arcagni P., Picard P. (2018). Efficacy and safety of a T-type calcium channel blocker in patients with neuropathic pain: A proof-of-concept, randomized, double-blind and controlled trial. Eur. J. Pain.

[80] Olsen R.W., DeLorey T.M., Siegel G.J., Agranoff B.W., Albers R.W., Fisher S.K., Uhler M.D. (1999). GABA and glycine. Basic Neurochemistry: Molecular, Cellular and Medical Aspects.

[81] Martin I.L., Dunn S.M. (2002). GABA receptors. Tocris. Rev..

[82] Malan T.P., Mata H.P., Porreca F. (2002). Spinal GABA(A) and GABA(B) receptor pharmacology in a rat model of neuropathic pain. Anesthesiology.

[83] Hwang J.H., Yaksh T.L. (1997). The effect of spinal GABA receptor agonists on tactile allodynia in a surgically-induced neuropathic pain model in the rat. Pain.

[84] Chen L., Wang W., Tan T., Han H., Dong Z. (2016). GABA(A) Receptors in the Central Nucleus of the Amygdala Are Involved in Pain- and Itch-Related Responses. J. Pain..

[85] Knabl J., Witschi R., Hösl K., Reinold H., Zeilhofer U.B., Ahmadi S., Brockhaus J., Sergejeva M., Hess A., Brune K. (2008). Reversal of pathological pain through specific spinal GABAA receptor subtypes. Nature.

[86] Atack J.R., Bayley P.J., Seabrook G.R., Wafford K.A., McKernan R.M., Dawson G.R. (2006). L-655,708 enhances cognition in rats but is not proconvulsant at a dose selective for alpha5-containing GABAA receptors. Neuropharmacology.

[87] Rivas F.M., Stables J.P., Murphree L., Edwankar R.V., Edwankar C.R., Huang S., Jain H.D., Zhou H., Majumder S., Sankar S. (2009). Antiseizure activity of novel gamma-aminobutyric acid (A) receptor subtype-selective benzodiazepine analogues in mice and rat models. J. Med. Chem..

[88] Di Lio A., Benke D., Besson M., Desmeules J., Daali Y., Wang Z.J., Edwankar R., Cook J.M., Zeilhofer H.U. (2011). HZ166, a novel GABAA receptor subtype-selective benzodiazepine site ligand, is antihyperalgesic in mouse models of inflammatory and neuropathic pain. Neuropharmacology.

[89] Griebel G., Perrault G., Simiand J., Cohen C., Granger P., Depoortere H., Françon D., Avenet P., Schoemaker H., Evanno Y. (2003). SL651498, a GABAA receptor agonist with subtype-selective efficacy, as a potential treatment for generalized anxiety disorder and muscle spasms. CNS Drug Rev..

[90] Matsubara M., Suzuki S., Miura K., Terashima M., Hatsuda S., Sugita S., Murakami H., Nakazawa K., Ohara M. (1997). A study of the effects of antidepressants on the GABAA receptor and its complex based on the drug actions on the power-spectral changes of rat hippocampal EEG induced by GABA antagonists and inverse agonists. Nihon Shinkei Seishin Yakurigaku Zasshi.

[91] Zheng T., Clarke A.L., Morris M.J., Reid C.A., Petrou S., O’Brien T.J. (2009). Oxcarbazepine, not its active metabolite, potentiates GABAA activation and aggravates absence seizures. Epilepsia.

[92] Lee P.R., Yoon S.Y., Kim H.W., Yeo J.H., Kim Y.H., Oh S.B. (2018). Peripheral GABAA receptor-mediated signaling facilitates persistent inflammatory hypersensitivity. Neuropharmacology.

[93] Vasović D., Divović B., Treven M., Knutson D.E., Steudle F., Scholze P., Obradović A., Fabjan J., Brković B., Sieghart W. (2019). Trigeminal neuropathic pain development and maintenance in rats are suppressed by a positive modulator of α6 GABAA receptors. Eur. J. Pain.

[94] De la Luz-Cuellar Y.E., Rodríguez-Palma E.J., Franco-Enzástiga Ú., Salinas-Abarca A.B., Delgado-Lezama R., Granados-Soto V. (2019). Blockade of spinal α5-GABAA receptors differentially reduces reserpine-induced fibromyalgia-type pain in female rats. Eur. J. Pharmacol..

[95] Masiulis S., Desai R., Uchański T., Serna Martin I., Laverty D., Karia D., Malinauskas T., Zivanov J., Pardon E., Kotecha A. (2019). GABAA receptor signalling mechanisms revealed by structural pharmacology. Nature.

[96] Goldstein A., Lowney L.I., Pal B.K. (1971). Stereospecific and Nonspecific Interactions of the Morphine Congener Levorphanol in Subcellular Fractions of Mouse Brain. Proc. Natl. Acad. Sci. USA.

[97] Janecka A., Fichna J., Janecki T. (2004). Opioid receptors and their ligands. Curr. Top. Med. Chem..

[98] Kieffer B.L., Gavériaux-Ruff C. (2002). Exploring the opioid system by gene knockout. Prog. Neurobiol..

[99] Law P.Y., Wong Y.H., Loh H.H. (2000). Molecular mechanisms and regulation of opioid receptor signaling. Annu. Rev. Pharmacol. Toxicol..

[100] Rosenblum A., Marsch L.A., Joseph H., Portenoy R.K. (2008). Opioids and the treatment of chronic pain: Controversies, current status, and future directions. Exp. Clin. Psychopharmacol..

[101] Nagar V.R., Birthi P., Salles S., Sloan P.A. (2017). Opioid Use in Chronic Pain Patients with Chronic Kidney Disease: A Systematic Review. Pain Med..

[102] Volkow N., Benveniste H., McLellan A.T. (2018). Use and Misuse of Opioids in Chronic Pain. Annu. Rev. Med..

[103] Benyamin R., Trescot A.M., Datta S., Buenaventura R., Adlaka R., Sehgal N., Glaser S.E., Vallejo R. (2008). Opioid complications and side effects. Pain Physician.

[104] Machelska H., Celik M.Ö. (2018). Advances in Achieving Opioid Analgesia Without Side Effects. Front. Pharmacol..

[105] Ehrlich A.T., Kieffer B.L., Darcq E. (2019). Current strategies toward safer mu opioid receptor drugs for pain management. Expert Opin. Ther. Targets.

[106] Tiwari V., Yang F., He S.Q., Shechter R., Zhang C., Shu B., Zhang T., Tiwari V., Wang Y., Dong X. (2016). Activation of Peripheral μ-opioid Receptors by Dermorphin [D-Arg2, Lys4] (1-4) Amide Leads to Modality-preferred Inhibition of Neuropathic Pain. Anesthesiology.

[107] Edwards K.A., Havelin J.J., Mcintosh M.I., Ciccone H.A., Pangilinan K., Imbert I., Largent-Milnes T.M., King T., Vanderah T.W., Streicher J.M. (2018). A Kappa Opioid Receptor Agonist Blocks Bone Cancer Pain Without Altering Bone Loss, Tumor Size, or Cancer Cell Proliferation in a Mouse Model of Cancer-Induced Bone Pain. J. Pain.

[108] Yi S.P., Kong Q.H., Li Y.L., Pan C.L., Yu J., Cui B.Q., Wang Y.F., Wang G.L., Zhou P.L., Wang L.L. (2017). The opioid receptor triple agonist DPI-125 produces analgesia with less respiratory depression and reduced abuse liability. Acta Pharmacol. Sin..

[109] Anand J.P., Kochan K.E., Nastase A.F., Montgomery D., Griggs N.W., Traynor J.R., Mosberg H.I., Jutkiewicz E.M. (2018). In vivo effects of μ-opioid receptor agonist/δ-opioid receptor antagonist peptidomimetics following acute and repeated administration. Br. J. Pharmacol..

[110] Minervini V., Lu H.Y., Padarti J., Osteicoechea D.C., France C.P. (2018). Interactions between kappa and mu opioid receptor agonists: Effects of the ratio of drugs in mixtures. Psychopharmacology.

[111] Spahn V., Del Vecchio G., Rodriguez-Gaztelumendi A., Temp J., Labuz D., Kloner M., Reidelbach M., Machelska H., Weber M., Stein C. (2018). Opioid receptor signaling, analgesic and side effects induced by a computationally designed pH-dependent agonist. Sci. Rep..

[112] Howlett A.C. (2002). The cannabinoid receptors. Prostaglandins Other Lipid Mediat..

[113] Aizpurua-Olaizola O., Elezgarai I., Rico-Barrio I., Zarandona I., Etxebarria N., Usobiaga A. (2017). Targeting the endocannabinoid system: Future therapeutic strategies. Drug. Discov. Today.

[114] Matsuda L.A., Lolait S.J., Brownstein M.J., Young A.C., Bonner T.I. (1990). Structure of a cannabinoid receptor and functional expression of the cloned cDNA. Nature.

[115] Yang L., Li Y., Ren J., Zhu C., Fu J., Lin D., Qiu Y. (2014). Celastrol attenuates inflammatory and neuropathic pain mediated by cannabinoid receptor type 2. Int. J. Mol. Sci..

[116] Toth C.C., Jedrzejewski N.M., Ellis C.L., Frey W.H. (2010). Cannabinoid-mediated modulation of neuropathic pain and microglial accumulation in a model of murine type I diabetic peripheral neuropathic pain. Mol. Pain.

[117] Deng L., Cornett B.L., Mackie K., Hohmann A.G. (2015). CB1 Knockout Mice Unveil Sustained CB2-Mediated Antiallodynic Effects of the Mixed CB1/CB2 Agonist CP55,940 in a Mouse Model of Paclitaxel-Induced Neuropathic Pain. Mol. Pharmacol..

[118] Heimann A.S., Gomes I., Dale C.S., Pagano R.L., Gupta A., de Souza L.L., Luchessi A.D., Castro L.M., Giorgi R., Rioli V. (2007). Hemopressin is an inverse agonist of CB1 cannabinoid receptors. Proc. Natl. Acad. Sci. USA.

[119] Lynch M.E., Cesar-Rittenberg P., Hohmann A.G. (2014). A double-blind, placebo-controlled, crossover pilot trial with extension using an oral mucosal cannabinoid extract for treatment of chemotherapy-induced neuropathic pain. J. Pain Symptom Manag..

[120] Vanhoenacker P., Haegeman G., Leysen J.E. (2000). 5-HT₇ receptors: Current knowledge and future prospects. Trends Pharmacol. Sci..

[121] Hedlund P.B., Sutcliffe J.G. (2004). Functional, molecular and pharmacological advances in 5-HT₇ receptor research. Trends Pharmacol. Sci..

[122] Hedlund P.B., Huitron-Resendiz S., Henriksen S.J., Sutcliffe J.G. (2005). 5-HT7 receptor inhibition and inactivation induce antidepressant like behavior and sleep pattern. Biol. Psychiatry.

[123] Naumenko V.S., Popova N.K., Lacivita E., Leopoldo M., Ponimaskin E.G. (2014). Interplay between Serotonin 5-HT1A and 5-HT₇ Receptors in Depressive Disorders. CNS Neurosci. Ther..

[124] Brenchat A., Romero L., García M., Pujol M., Burgueño J., Torrens A., Hamon M., Baeyens J.M., Buschmann H., Zamanillo D. (2009). 5-HT7 receptor activation inhibits mechanical hypersensitivity secondary to capsaicin sensitization in mice. Pain.

[125] Brenchat A., Nadal X., Romero L., Ovalle S., Muro A., Sánchez-Arroyos R., Portillo-Salido E., Pujol M., Montero A., Codony X. (2010). Pharmacological activation of 5-HT7 receptors reduces nerve injury-induced mechanical and thermal hypersensitivity. Pain.

[126] Demirkaya K., Akgün Ö.M., Şenel B., Öncel Torun Z., Seyrek M., Lacivita E., Leopoldo M., Doğrul A. (2016). Selective 5-HT7 receptor agonists LP 44 and LP 211 elicit an analgesic effect on formalin-induced orofacial pain in mice. J. Appl. Oral Sci..

[127] Santello M., Bisco A., Nevian N.E., Lacivita E., Leopoldo M., Nevian T. (2017). The brain-penetrant 5-HT7 receptor agonist LP-211 reduces the sensory and affective components of neuropathic pain. Neurobiol. Dis..

[128] Lax N., Hilton E., Ahmed T., Tidgewell K., Kolber B. (2017). Understanding the role of serotonin receptor subtype 7 (5-HT7) in comorbid pain and depression using novel compounds derived from marine cyanobacteria. J. Pain.

[129] Zhang J.M., An J. (2007). Cytokines, inflammation, and pain. Int. Anesthesiol. Clin..

[130] Blaustein M.P., Lederer W.J. (1999). Sodium/calcium exchange: Its physiological implications. Physiol. Rev..

[131] Annunziato L., Pignataro G., Di Renzo G.F. (2004). Pharmacology of Brain Na/Ca-Exchanger: From Molecular Biology to Therapeutic Perspectives. Pharmacol. Rev..

[132] Lavoie P.A., Beauchamp G., Elie R. (1990). Tricyclic antidepressants inhibit voltage-dependent calcium channels and Na(+)-Ca2+ exchange in rat brain cortex synaptosomes. Can. J. Physiol. Pharmacol..

[133] Iwamoto T., Watanabe Y., Kita S., Blaustein M.P. (2007). Na+/Ca2+ exchange inhibitors: A new class of calcium regulators. Cardiovasc. Hematol. Disord. Drug Targets.

[134] Datta S., Waghray T., Torres M., Glusman S. (2004). Amiodarone decreases heat, cold, and mechanical hyperalgesia in a rat model of neuropathic pain. Anesth. Analg..

[135] Jaggi A.S., Singh N. (2011). Therapeutic targets for the management of peripheral nerve injury-induced neuropathic pain. CNS Neurol. Disord. Drug Targets.

[136] Sibarov D.A., Abushik P.A., Poguzhelskaya E.E., Bolshakov K.V., Antonov S.M. (2015). Inhibition of plasma membrane Na/Ca-exchanger by KB-R7943 or lithium reveals its role in Ca-dependent N-methyl-D-aspartate receptor inactivation. J. Pharmacol. Exp. Ther..

[137] Sibarov D.A., Poguzhelskaya E.E., Antonov S.M. (2018). Downregulation of calcium-dependent NMDA receptor desensitization by sodium-calcium exchangers: A role of membrane cholesterol. BMC Neurosci..

[138] Liu T., Ji R.R. (2013). New insights into the mechanisms of itch: Are pain and itch controlled by distinct mechanisms?. Pflugers Arch..

[139] Lee J.S., Han J.S., Lee K., Bang J., Lee H. (2016). The peripheral and central mechanisms underlying itch. BMB Rep..

[140] Hill S.J., Ganellin C.R., Timmerman H., Schwartz J.C., Shankley N.P., Young J.M., Schunack W., Levi R., Haas H.L. (1997). International Union of Pharmacology. XIII. Classification of histamine receptors. Pharmacol. Rev..

[141] Parsons M.E., Ganellin C.R. (2006). Histamine and its receptors. Br. J. Pharmacol..

[142] Rossbach K., Nassenstein C., Gschwandtner M., Schnell D., Sander K., Seifert R., Stark H., Kietzmann M., Bäumer W. (2011). Histamine H1, H3 and H4 receptors are involved in pruritus. Neuroscience.

[143] Ohsawa Y., Hirasawa N. (2014). The role of histamine H1 and H4 receptors in atopic dermatitis: From basic research to clinical study. Allergol. Int..

[144] Dhand A., Aminoff M.J. (2014). The neurology of itch. Brain.

[145] O’Donoghue M., Tharp M.D. (2005). Antihistamines and their role as antipruritics. Dermatol Ther..

[146] Borowy C.S., Mukherji P. (2018). Antihistamine Toxicity.

[147] Procopiou P.A., Ford A.J., Gore P.M., Looker B.E., Hodgson S.T., Holmes D.S., Vile S., Clark K.L., Saunders K.A., Slack R.J. (2017). Design of Phthalazinone Amide Histamine H1 Receptor Antagonists for Use in Rhinitis. ACS Med. Chem. Lett..

[148] Ledneczki I., Tapolcsányi P., Gábor E., Éles J., Greiner I., Schmidt É., Némethy Z., Kedves R.S., Balázs O., Román V. (2017). Discovery of novel steroidal histamine H3 receptor antagonists/inverse agonists. Bioorg. Med. Chem. Lett..

[149] Ko K., Kim H.J., Ho P.S., Lee S.O., Lee J.E., Min C.R., Kim Y.C., Yoon J.H., Park E.J., Kwon Y.J. (2018). Discovery of a Novel Highly Selective Histamine H4 Receptor Antagonist for the Treatment of Atopic Dermatitis. J. Med. Chem..

[150] Li M., Yu Y., Yang J. (2011). Structural biology of TRP channels. Adv. Exp. Med. Biol..

[151] Basso L., Altier C. (2017). Transient Receptor Potential Channels in neuropathic pain. Curr. Opin. Pharmacol..

[152] Moran M.M., Szallasi A. (2018). Targeting nociceptive transient receptor potential channels to treat chronic pain: Current state of the field. Br. J. Pharmacol..

[153] Xie Z., Hu H. (2018). TRP Channels as Drug Targets to Relieve Itch. Pharmaceuticals.

[154] Kittaka H., Yamanoi Y., Tominaga M. (2017). Transient receptor potential vanilloid 4 (TRPV4) channel as a target of crotamiton and its bimodal effects. Pflugers Arch..

[155] Akiyama T., Ivanov M., Nagamine M., Davoodi A., Carstens M.I., Ikoma A., Cevikbas F., Kempkes C., Buddenkotte J., Steinhoff M. (2016). Involvement of TRPV4 in Serotonin-Evoked Scratching. J. Investig. Dermatol..

[156] Chen Y., Fang Q., Wang Z., Zhang J.Y., MacLeod A.S., Hall R.P., Liedtke W.B. (2016). Transient Receptor Potential Vanilloid 4 Ion Channel Functions as a Pruriceptor in Epidermal Keratinocytes to Evoke Histaminergic Itch. J. Biol. Chem..

[157] Luo J., Feng J., Yu G., Yang P., Mack M.R., Du J., Yu W., Qian A., Zhang Y., Liu S. (2018). Transient receptor potential vanilloid 4-expressing macrophages and keratinocytes contribute differentially to allergic and nonallergic chronic itch. J. Allergy Clin. Immunol..

[158] Sawynok J. (2016). Adenosine receptor targets for pain. Neuroscience.

[159] Alves L.A., Bezerra R.J., Faria R.X., Ferreira L.G., da Silva Frutuoso V. (2013). Physiological roles and potential therapeutic applications of the P2 × 7 receptor in inflammation and pain. Molecules.

[160] Obata H. (2017). Analgesic Mechanisms of Antidepressants for Neuropathic Pain. Int. J. Mol. Sci..

[161] Busserolles J., Tsantoulas C., Eschalier A., López García J.A. (2016). Potassium channels in neuropathic pain: Advances, challenges, and emerging ideas. Pain.

[162] Wang J., Casals-Diaz L., Zurawski T., Meng J., Moriarty O., Nealon J., Edupuganti O.P., Dolly O. (2017). A novel therapeutic with two SNAP-25 inactivating proteases shows long-lasting anti-hyperalgesic activity in a rat model of neuropathic pain. Neuropharmacology.

[163] Kim W.M., Lee S.H., Jeong H.J., Lee H.G., Choi J.I., Yoon M.H. (2012). The analgesic activity of intrathecal tianeptine, an atypical antidepressant, in a rat model of inflammatory pain. Anesth. Analg..

[164] Heo B.H., Shin J.Y., Park K.S., Lee H.G., Choi J.I., Yoon M.H., Kim W.M. (2016). Effects of tianeptine on the development and maintenance of mechanical allodynia in a rat model of neuropathic pain. Neurosci. Lett..

[165] Han S.M., Kim Y.H., Jo H.U., Kwak J.A., Park H.J. (2017). Tianeptine Reduces Mechanical Allodynia in Spinal Nerve-ligated and Chemotherapy-induced Neuropathic Mice. Pain Physician.

[166] Lee H., Im J., Won H., Nam W., Kim Y.O., Lee S.W., Lee S., Cho I.H., Kim H.K., Kwon J.T. (2017). Effects of tianeptine on symptoms of fibromyalgia via BDNF signaling in a fibromyalgia animal model. Korean J. Physiol. Pharmacol..

[167] El Zahran T., Schier J., Glidden E., Kieszak S., Law R., Bottei E., Aaron C., King A., Chang A. (2018). Characteristics of Tianeptine Exposures Reported to the National Poison Data System—United States, 2000–2017. MMWR.

[168] Svenningsson P., Bateup H., Qi H., Takamiya K., Huganir R.L., Spedding M., Roth B.L., McEwen B.S., Greengard P. (2007). Involvement of AMPA receptor phosphorylation in antidepressant actions with special reference to tianeptine. Eur. J. Neurosci..

[169] Gassaway M.M., Rives M.L., Kruegel A.C., Javitch J.A., Sames D. (2014). The atypical antidepressant and neurorestorative agent tianeptine is a μ-opioid receptor agonist. Transl. Psychiatry.

[170] Bilge S.S., İlkaya F., Darakcı Ö., Çiftcioğlu E., Bozkurt A. (2018). Opioid Receptors Contribute to Antinociceptive Effect of Tianeptine on Colorectal Distension-Induced Visceral Pain in Rats. Pharmacology.

[171] Lin H., Heo B.H., Kim W.M., Kim Y.C., Yoon M.H. (2015). Antiallodynic effect of tianeptine via modulation of the 5-HT7 receptor of GABAergic interneurons in the spinal cord of neuropathic rats. Neurosci. Lett..

[172] Kato G., Weitsch A.F. (1988). Neurochemical profile of tianeptine, a new antidepressant drug. Clin. Neuropharmacol..

[173] Ardid D., Marty H., Fialip J., Privat A.M., Eschalier A., Lavarenne J. (1992). Comparative effects of different uptake inhibitor antidepressants in two pain tests in mice. Fundam. Clin. Pharmacol..

[174] Bomholt S.F., Mikkelsen J.D., Blackburn-Munro G. (2005). Antinociceptive effects of the antidepressants amitriptyline, duloxetine, mirtazapine and citalopram in animal models of acute, persistent and neuropathic pain. Neuropharmacology.

[175] Sindrup S.H., Bjerre U., Dejgaard A., Brøsen K., Aaes-Jørgensen T., Gram L.F. (1992). The selective serotonin reuptake inhibitor citalopram relieves the symptoms of diabetic neuropathy. Clin. Pharmacol. Ther..

[176] Nørregaard J., Volkmann H., Danneskiold-Samsøe B. (1995). A randomized controlled trial of citalopram in the treatment of fibromyalgia. Pain.

[177] Bendtsen L., Jensen R., Olesen J. (1996). A non-selective (amitriptyline), but not a selective (citalopram), serotonin reuptake inhibitor is effective in the prophylactic treatment of chronic tension-type headache. J. Neurol. Neurosurg. Psychiatry.

[178] Anderberg U.M., Marteinsdottir I., von Knorring L. (2000). Citalopram in patients with fibromyalgia—A randomized, double-blind, placebo-controlled study. Eur. J. Pain.

[179] Aragona M., Bancheri L., Perinelli D., Tarsitani L., Pizzimenti A., Conte A., Inghilleri M. (2005). Randomized double-blind comparison of serotonergic (Citalopram) versus noradrenergic (Reboxetine) reuptake inhibitors in outpatients with somatoform, DSM-IV-TR pain disorder. Eur. J. Pain.

[180] Giannopoulos S., Kosmidou M., Sarmas I., Markoula S., Pelidou S.H., Lagos G., Kyritsis A.P. (2007). Patient compliance with SSRIs and gabapentin in painful diabetic neuropathy. Clin. J. Pain.

[181] D’Erme A.M., Zanieri F., Campolmi E., Santosuosso U., Betti S., Agnoletti A.F., Cossidente A., Lotti T. (2014). Therapeutic implications of adding the psychotropic drug escitalopram in the treatment of patients suffering from moderate-severe psoriasis and psychiatric comorbidity: A retrospective study. J. Eur. Acad. Dermatol. Venereol..

[182] Thériault O., Poulin H., Beaulieu J.M., Chahine M. (2015). Differential modulation of Nav1.7 and Nav1.8 channels by antidepressant drugs. Eur. J. Pharmacol..

[183] Witchel H.J., Pabbathi V.K., Hofmann G., Paul A.A., Hancox J.C. (2002). Inhibitory actions of the selective serotonin re-uptake inhibitor citalopram on HERG and ventricular L-type calcium currents. FEBS Lett..

[184] Hamplová-Peichlová J., Krůsek J., Paclt I., Slavícek J., Lisá V., Vyskocil F. (2002). Citalopram Inhibits L-type Calcium Channel Current in Rat Cardiomyocytes in Culture. Physiol. Res..

[185] Zahradník I., Minarovic I., Zahradníková A. (2008). Inhibition of the cardiac L-type calcium channel current by antidepressant drugs. J. Pharmacol. Exp. Ther..

[186] Hyttel J. (1982). Citalopram—Pharmacological profile of a specific serotonin uptake inhibitor with antidepressant activity. Prog. Neuropsychopharmacol. Biol. Psychiatry.

[187] Hesketh S.A., Brennan A.K., Jessop D.S., Finn D.P. (2008). Effects of chronic treatment with citalopram on cannabinoid and opioid receptor-mediated G-protein coupling in discrete rat brain regions. Psychopharmacology.

[188] Owens M.J., Knight D.L., Nemeroff C.B. (2001). Second-generation SSRIs: Human monoamine transporter binding profile of escitalopram and R-fluoxetine. Biol. Psychiatry.

[189] Zarrindast M.R., Alaei-Nia K., Shafizadeh M. (2001). On the mechanism of tolerance to morphine-induced Straub tail reaction in mice. Pharmacol. Biochem. Behav..

[190] Pakulska W., Czarnecka E. (2002). Influence of mianserin on the antinociceptive effect of morphine, metamizol and indomethacin in mice. Pharmacol. Res..

[191] Üçel U.İ., Can Ö.D., Demir Özkay Ü., Öztürk Y. (2015). Antihyperalgesic and antiallodynic effects of mianserin on diabetic neuropathic pain: A study on mechanism of action. Eur. J. Pharmacol..

[192] Berendsen H.H.G., Broekkamp C.L.E. (1991). A peripheral 5-HT1D-like receptor involved in serotonergic induced hindlimb scratching in rats. Eur. J. Pharmacol..

[193] Langemark M., Loldrup D., Bech P., Olesen J. (1990). Clomipramine and mianserin in the treatment of chronic tension headache. A double-blind, controlled study. Headache.

[194] Van Houdenhove B., Verstraeten D., Onghena P., De Cuyper H. (1992). Chronic idiopathic pain, mianserin and ‘masked’ depression. Psychother. Psychosom..

[195] Onghena P., De Cuyper H., Van Houdenhove B., Verstraeten D. (1993). Mianserin and chronic pain: A double-blind placebo-controlled process and outcome study. Acta Psychiatr. Scand..

[196] Manna V., Bolino F., Di Cicco L. (1994). Chronic tension-type headache, mood depression and serotonin: Therapeutic effects of fluvoxamine and mianserin. Headache.

[197] Tohda M., Urushihara H., Nomura Y. (1995). Inhibitory effects of antidepressants on NMDA-induced currents in Xenopus oocytes injected with rat brain RNA. Neurochem. Int..

[198] Lenkey N., Karoly R., Lukacs P., Vizi E.S., Sunesen M., Fodor L., Mike A. (2010). Classification of drugs based on properties of sodium channel inhibition: A comparative automated patch-clamp study. PLoS ONE.

[199] Lazar A., Lenkey N., Pesti K., Fodor L., Mike A. (2015). Different pH-sensitivity patterns of 30 sodium channel inhibitors suggest chemically different pools along the access pathway. Front. Pharmacol..

[200] Becker B., Morel N., Vanbellinghen A.M., Lebrun P. (2004). Blockade of calcium entry in smooth muscle cells by the antidepressant imipramine. Biochem. Pharmacol..

[201] Boselli C., Santagostino-Barbone M., Lucchelli A. (2007). Older versus newer antidepressants: Substance P or calcium antagonism?. Can. J. Physiol. Pharmacol..

[202] Blier P., de Montigny C., Tardif D. (1984). Effects of the two antidepressant drugs mianserin and indalpine on the serotonergic system: Single-cell studies in the rat. Psychopharmacology.

[203] Olianas M.C., Dedoni S., Onali P. (2012). The atypical antidepressant mianserin exhibits agonist activity at κ-opioid receptors. Br. J. Pharmacol..

[204] Schreiber S., Backer M.M., Kaufman J.P., Pick C.G. (1998). Interaction between the tetracyclic antidepressant mianserin HCl and opioid receptors. Eur. Neuropsychopharmacol..

[205] Lucchelli A., Santagostino-Barbone M.G., D’Agostino G., Masoero E., Tonini M. (2000). The interaction of antidepressant drugs with enteric 5-HT7 receptors. Naunyn-Schmiedeberg’s Arch. Pharmacol..

[206] Röser C., Jordan N., Balfanz S., Baumann A., Walz B., Baumann O., Blenau W. (2012). Pharmacological Characterization of Serotonin 5-HT2α and 5-HT7 Receptors in the Salivary Glands of the Blowfly Calliphora vicina. PLoS ONE.

[207] Hall H., Ogren S.O. (1984). Effects of antidepressant drugs on histamine-H1 receptors in the brain. Life Sci..

[208] Kanba S., Richelson E. (1984). Histamine H1 receptors in human brain labelled with [3H]doxepin. Brain Res..

[209] Nguyen T., Shapiro D.A., George S.R., Setola V., Lee D.K., Cheng R., Rauser L., Lee S.P., Lynch K.R., Roth B.L. (2001). Discovery of a novel member of the histamine receptor family. Mol. Pharmacol..

[210] Bianchi M., Rossoni G., Sacerdote P., Panerai A.E., Berti F. (1995). Carbamazepine exerts anti-inflammatory effects in the rat. Eur. J. Pharmacol..

[211] Sakaue A., Honda M., Tanabe M., Ono H. (2004). Antinociceptive effects of sodium channel-blocking agents on acute pain in mice. J. Pharmacol. Sci..

[212] Aoki M., Tsuji M., Takeda H., Harada Y., Nohara J., Matsumiya T., Chiba H. (2006). Antidepressants enhance the antinociceptive effects of carbamazepine in the acetic acid-induced writhing test in mice. J. Pharmacol..

[213] Chogtu B., Bairy K.L., Smitha D., Dhar S., Himabindu P. (2011). Comparison of the efficacy of carbamazepine, gabapentin and lamotrigine for neuropathic pain in rats. Indian J. Pharmacol..

[214] Mohajjel Nayebi A., Sharifi H., Ramadzani M., Rezazadeh H. (2012). Effect of acute and chronic administration of carbamazepine on Cisplatin-induced hyperalgesia in rats. Jundishapur J. Nat. Pharm. Prod..

[215] Hama A.T., Pearson J.P., Sagen J. (2014). Effects of repeated dosing with mechanistically distinct antinociceptive ligands in a rat model of neuropathic spinal cord injury pain. Pharmacol. Res. Perspect..

[216] Deseure K., Hans G.H. (2017). Differential drug effects on spontaneous and evoked pain behavior in a model of trigeminal neuropathic pain. J. Pain Res..

[217] Campbell F.G., Graham J.G., Zilkha K.J. (1966). Clinical trial of carbazepine (tegretol) in trigeminal neuralgia. J. Neurol. Neurosurg. Psychiatry.

[218] Taylor J.C., Brauer S., Espir M.L. (1981). Long-term treatment of trigeminal neuralgia with carbamazepine. Postgrad. Med. J..

[219] Qi L., Liu C., Huang M. A double-blind study of the effect of phenytoin on trigeminal neuralgia. Presented at the National Workshop of Clinical Use of Phenytoin, Chengdu, China, 1995, 3225. http://www.remarkablemedicine.com/Clinical/clinicaluses/paintreatment/trigeminal.html.

[220] Wilton T.D. (1974). Tegretol in the treatment of diabetic neuropathy. S. Afr. Med. J..

[221] Saeed T., Nasrullah M., Ghafoor A., Shahid R., Islam N., Khattak M.U., Maheshwary N., Siddiqi A., Khan M.A. (2014). Efficacy and tolerability of carbamazepine for the treatment of painful diabetic neuropathy in adults: A 12-week, open-label, multicenter study. Int. J. Gen. Med..

[222] Yamamoto M., Yabuki S., Hayabara T., Otsuki S. (1981). Paroxysmal itching in multiple sclerosis: A report of three cases. J. Neurol. Neurosurg. Psychiatry.

[223] Tait C.P., Grigg E., Quirk C.J. (1998). Brachioradial pruritus and cervical spine manipulation. Australas. J. Dermatol..

[224] Bhushan M., Parry E.J., Telfer N.R. (1999). Trigeminal trophic syndrome: Successful treatment with carbamazepine. Br. J. Dermatol..

[225] Basselin M., Villacreses N.E., Chen M., Bell J.M., Rapoport S.I. (2007). Chronic carbamazepine administration reduces N-methyl-D-aspartate receptor-initiated signaling via arachidonic acid in rat brain. Biol. Psychiatry.

[226] Rezvanfard M., Zarrindast M.R., Bina P. (2009). Role of ventral hippocampal GABA(A) and NMDA receptors in the anxiolytic effect of carbamazepine in rats using the elevated plus maze test. Pharmacology.

[227] Matsumoto A., Arisaka H., Hosokawa Y., Sakuraba S., Sugita T., Umezawa N., Kaku Y., Yoshida K., Kuwana S. (2015). Effect of carbamazepine and gabapentin on excitability in the trigeminal subnucleus caudalis of neonatal rats using a voltage-sensitive dye imaging technique. Biol. Res..

[228] Willow M., Gonoi T., Catterall W.A. (1985). Voltage-clamp analysis of the inhibitory actions of diphenylhydantoin and carbamazepine on voltage-sensitive sodium channels in neuroblastoma cells. Mol. Pharmacol..

[229] McLean M.J., Macdonald R.L. (1986). Carbamazepine and 10,11-epoxycarbamazepine produce use- and voltage-dependent limitation of rapidly firing action potentials of mouse central neurons in cell culture. J. Pharmacol. Exp. Ther..

[230] Ragsdale S., Scheuer T., Catterall W.A. (1994). Frequency and voltage-dependent inhibition of type IIA Na channels, expressed in a mammalian cell line, by local anesthetic, antiarrhythmic and anticonvulsant drugs. Mol. Pharmacol..

[231] Kuo C.C., Chen R.S., Lu L., Chen R.C. (1997). Carbamazepine inhibition of neuronal Na currents: Quantitative distinction from phenytoin and possible therapeutic implications. Mol. Pharmacol..

[232] Jo S., Bean B.P. (2014). Sidedness of carbamazepine accessibility to voltage-gated sodium channels. Mol. Pharmacol..

[233] Walden J., Grunze H., Bingmann D., Liu Z., Düsing R. (1992). Calcium antagonistic effects of carbamazepine as a mechanism of action in neuropsychiatric disorders: Studies in calcium dependent model epilepsies. Eur. Neuropsychopharmacol..

[234] Schumacher T.B., Beck H., Steinhäuser C., Schramm J., Elger C.E. (1998). Effects of phenytoin, carbamazepine, and gabapentin on calcium channels in hippocampal granule cells from patients with temporal lobe epilepsy. Epilepsia.

[235] Granger P., Biton B., Faure C., Vige X., Depoortere H., Graham D., Langer S.Z., Scatton B., Avenet P. (1995). Modulation of the gamma-aminobutyric acid type A receptor by the antiepileptic drugs carbamazepine and phenytoin. Mol. Pharmacol..

[236] Jackson H.C., Nutt D.J. (1993). Investigation of the involvement of opioid receptors in the action of anticonvulsants. Psychopharmacology.

[237] Due M.R., Yang X.-F., Allette Y.M., Randolph A.L., Ripsch M.S., Wilson S.M., Dustrude E.T., Khanna R., White F.A. (2014). Carbamazepine Potentiates the Effectiveness of Morphine in a Rodent Model of Neuropathic Pain. PLoS ONE.

[238] Siniscalchi A., Gallelli L., Avenoso T., Squillace A., De Sarro G. (2011). Effects of carbamazepine/oxycodone coadministration in the treatment of trigeminal neuralgia. Ann. Pharmacother..

[239] Tomić M.A., Pecikoza U.B., Micov A.M., Stepanović-Petrović R.M. (2015). The Efficacy of Eslicarbazepine Acetate in Models of Trigeminal, Neuropathic, and Visceral Pain: The Involvement of 5-HT1B/1D Serotonergic and CB1/CB2 Cannabinoid Receptors. Anesth. Analg..

[240] Oláh Z., Jósvay K., Pecze L., Letoha T., Babai N., Budai D., Otvös F., Szalma S., Vizler C. (2007). Anti-calmodulins and tricyclic adjuvants in pain therapy block the TRPV1 channel. PLoS ONE.

[241] Schreiber S., Backer M.M., Herman I., Shamir D., Boniel T., Pick C.G. (2000). The antinociceptive effect of trazodone in mice is mediated through both µ-opioid and serotonergic mechanisms. Behav. Brain Res..

[242] Okuda K., Takanishi T., Yoshimoto K., Ueda S. (2003). Trazodone hydrochloride attenuates thermal hyperalgesia in achronic constriction injury rat model. Eur. J. Anaesthesiol..

[243] Zhang R., Nagata T., Hayashi T., Miyata M., Kawakami Y. (2004). Intracerebroventricular injection of trazodone produces 5-HT receptor subtype mediated anti-nociception at the supraspinal and spinal levels. Eur. Neuropsychopharmacol..

[244] Wilson R.C. (1999). The use of low-dose trazodone in the treatment of painful diabetic neuropathy. J. Am. Podiatr. Med. Assoc..

[245] Morillas-Arques P., Rodriguez-Lopez C.M., Molina-Barea R., Rico-Villademoros F., Calandre E.P. (2010). Trazodone for the treatment of fibromyalgia: An open-label, 12-week study. BMC Musculoskelet. Disord..

[246] Calandre E.P., Morillas-Arques P., Molina-Barea R., Rodriguez-Lopez C.M., Rico-Villademoros F. (2011). Trazodone plus pregabalin combination in the treatment of fibromyalgia: A two-phase, 24-week, open-label uncontrolled study. BMC Musculoskelet. Disord..

[247] Pancrazio J.J., Kamatchi G.L., Roscoe A.K., Lynch C. (1998). Inhibition of neuronal Na+ channels by antidepressant drugs. J. Pharmacol. Exp. Ther..

[248] Lee S., Lee H.A., Kim S.J., Kim K.S. (2016). Cellular mechanisms for trazodone-induced cardiotoxicity. Hum. Exp. Toxicol..

[249] Kraus R.L., Li Y., Jovanovska A., Renger J.J. (2007). Trazodone inhibits T-type calcium channels. Neuropharmacology.

[250] Richelson E., Nelson A. (1984). Antagonism by antidepressants of neurotransmitter receptors of normal human brain in vitro. J. Pharmacol. Exp. Ther..

[251] Scholl B., Burge J., Priebe N.J. (2013). Binocular integration and disparity selectivity in mouse primary visual cortex. J. Neurophysiol..

[252] Tantirigama M.L., Huang H.H., Bekkers J.M. (2017). Spontaneous activity in the piriform cortex extends the dynamic range of cortical odor coding. Proc. Natl. Acad. Sci. USA.

[253] Jones R.K. (1974). Meralgia paresthetica as a cause of leg discomfort. Can. Med. Assoc. J..

[254] Farber G.A., Burks J.W. (1974). Chlorprothixene therapy for herpes zoster neuralgia. South Med. J..

[255] Nathan P.W. (1978). Chlorprothixene (taractan) in post-herpetic neuralgia and other severe chronic pains. Pain.

[256] Kramer P.W. (1981). The management of postherpetic neuralgia with chlorprothixene. Surg. Neurol..

[257] Squires R.F., Saederup E. (1991). A review of evidence for GABergic predominance/glutamatergic deficit as a common etiological factor in both schizophrenia and affective psychoses: More support for a continuum hypothesis of “functional” psychosis. Neurochem. Res..

[258] Squires R.F., Saederup E. (1998). Clozapine and several other antipsychotic/antidepressant drugs preferentially block the same ‘core’ fraction of GABA(A) receptors. Neurochem. Res..

[259] Roth B.L., Craigo S.C., Choudhary M.S., Uluer A., Monsma F.J., Shen Y., Meltzer H.Y., Sibley D.R. (1994). Binding of typical and atypical antipsychotic agents to 5-hydroxytryptamine-6 and 5-hydroxytryptamine-7 receptors. J. Pharmacol. Exp. Ther..

[260] Glusa E., Pertz H.H. (2000). Further evidence that 5-HT-induced relaxation of pig pulmonary artery is mediated by endothelial 5-HT(2B) receptors. Br. J. Pharmacol..

[261] Von Coburg Y., Kottke T., Weizel L., Ligneau X., Stark H. (2009). Potential utility of histamine H3 receptor antagonist pharmacophore in antipsychotics. Bioorg. Med. Chem. Lett..

[262] Acs G., Palkovits M., Blumberg P.M. (1995). Trifluoperazine modulates [3H]resiniferatoxin binding by human and rat vanilloid (capsaicin) receptors and affects 45Ca uptake by adult rat dorsal root ganglion neurones. J. Pharmacol. Exp. Ther..

[263] Marchand F., Alloui A., Chapuy E., Hernandez A., Pelissier T., Ardid D., Eschalier A. (2003). The antihyperalgesic effect of venlafaxine in diabetic rats does not involve the opioid system. Neurosci. Lett..

[264] Aricioğlu F., Buldanlioğlu U., Salanturoğlu G., Ozyalçin N.S. (2005). Evaluation of antinociceptive and anti-inflammatory effects of venlafaxine in the rat. AĞRI.

[265] Hajhashemi V., Minaiyan M., Banafshe H.R., Mesdaghinia A., Abed A. (2015). The anti-inflammatory effects of venlafaxine in the rat model of carrageenan-induced paw edema. Iran. J. Basic Med. Sci..

[266] Folkesson A., Honoré P.H., Bjerrum O.J. (2010). Co-administered gabapentin and venlafaxine in nerve injured rats: Effect on mechanical hypersensitivity, motor function and pharmacokinetics. Scand. J. Pain.

[267] Hajhashemi V., Banafshe H.R., Minaiyan M., Mesdaghinia A., Abed A. (2014). Antinociceptive effects of venlafaxine in a rat model of peripheral neuropathy: Role of alpha2-adrenergic receptors. Eur. J. Pharmacol..

[268] Mansouri M.T., Naghizadeh B., Ghorbanzadeh B., Alboghobeish S., Amirgholami N., Houshmand G., Cauli O. (2018). Venlafaxine prevents morphine antinociceptive tolerance: The role of neuroinflammation and the l-arginine-nitric oxide pathway. Exp. Neurol..

[269] Cegielska-Perun K., Tatarkiewicz J., Siwek A., Dybała M., Bujalska-Zadrożny M. (2015). Mechanisms of morphine–venlafaxine interactions in diabetic neuropathic pain model. Pharmacol. Rep..

[270] Rowbotham M.C., Goli V., Kunz N.R., Lei D. (2004). Venlafaxine extended release in the treatment of painful diabetic neuropathy: A double-blind, placebo-controlled study. Pain.

[271] Jia H.Y., Li Q.F., Song D.P., An Z.M., Liu Y.P., Ran X.W., Wu R.H., Tian H.M. (2006). Effect of venlafaxine and carbamazepine for painful peripheral diabetic neuropathy: A randomized, double-blind and double-dummy, controlled multi-center trial. Chin. J. Evid. Based Med..

[272] Piletz J.E., Halaris A., Iqbal O., Hoppensteadt D., Fareed J., Zhu H., Sinacore J., Devane C.L. (2009). Pro-inflammatory biomakers in depression: Treatment with venlafaxine. World J. Biol. Psychiatry.

[273] Lee H., Kim J.H., Min B.-H., Lee J.H., Son H.J., Kim J.J., Rhee P.-L. (2010). Efficacy of Venlafaxine for Symptomatic Relief in Young Adult Patients with Functional Chest Pain: A Randomized, Double-Blind, Placebo-Controlled, Crossover Trial. Am. J. Gastroenterol..

[274] Razazian N., Baziyar M., Moradian N., Afshari D., Bostani A., Mahmoodi M. (2014). Evaluation of the efficacy and safety of pregabalin, venlafaxine, and carbamazepine in patients with painful diabetic peripheral neuropathy. A randomized, double-blind trial. Neurosciences (Riyadh).

[275] Gallagher H.C., Gallagher R.M., Butler M., Buggy D.J., Henman M.C. (2015). Venlafaxine for neuropathic pain in adults. Cochrane Database Syst. Rev..

[276] Farshchian N., Alavi A., Heydarheydari S., Moradian N. (2018). Comparative study of the effects of venlafaxine and duloxetine on chemotherapy-induced peripheral neuropathy. Cancer Chemother. Pharmacol..

[277] Raabe R., Gentile L. (2008). Antidepressant interactions with the NMDA NR1-1b subunit. J. Biophys..

[278] Yilmaz N., Demirdas A., Yilmaz M., Sutcu R., Kirbas A., Cure M.C., Eren I. (2011). Effects of venlafaxine and escitalopram treatments on NMDA receptors in the rat depression model. J. Membr. Biol..

[279] Tamási V., Petschner P., Adori C., Kirilly E., Ando R.D., Tothfalusi L., Juhasz G., Bagdy G. (2014). Transcriptional evidence for the role of chronic venlafaxine treatment in neurotrophic signaling and neuroplasticity including also Glutamatergic- and insulin-mediated neuronal processes. PLoS ONE.

[280] Khalifa M., Daleau P., Turgeon A.J. (1999). Mechanism of sodium channel block by venlafaxine in guinea pig ventricular myocytes. J. Pharmacol. Exp. Ther..

[281] Schreiber S., Backer M.M., Pick C.G. (1999). The antinociceptive effect of venlafaxine in mice is mediated through opioid and adrenergic mechanisms. Neurosci. Lett..

[282] Sikka P., Kaushik S., Kumar G., Kapoor S., Bindra V.K., Saxena K.K. (2011). Study of antinociceptive activity of SSRI (fluoxetine and escitalopram) and atypical antidepressants (venlafaxine and mirtazepine) and their interaction with morphine and naloxone in mice. J. Pharm. Bioallied. Sci..

[283] Bymaster F.P., Dreshfield-Ahmad L.J., Threlkeld P.G., Shaw J.L., Thompson L., Nelson D.L., Hemrick-Luecke S.K., Wong D.T. (2001). Comparative affinity of duloxetine and venlafaxine for serotonin and norepinephrine transporters in vitro and in vivo, human serotonin receptor subtypes, and other neuronal receptors. Neuropsychopharmacology.

[284] Miskovic M. (2015). Comparison of tolerance of venlafaxine, paroxetine and amitriptyline in depression therapy. Med. Arch..

[285] Shen S., Zhang C., Mao Y. (2018). Determination of venlafaxine and its active metabolite O-desmethylvenlafaxine in human plasma by HPLC fluorescence. Gen. Psychiatr..

[286] Miyano K., Minami K., Yokoyama T., Ohbuchi K., Yamaguchi T., Murakami S., Shiraishi S., Yamamoto M., Matoba M., Uezono Y. (2015). Tramadol and its metabolite m1 selectively suppress transient receptor potential ankyrin 1 activity, but not transient receptor potential vanilloid 1 activity. Anesth. Analg..

[287] Plummer N.W., Meisler M.H. (1999). Evolution and diversity of mammalian sodium channel genes. Genomics.

[288] Brouwer B.A., Merkies I.S., Gerrits M.M., Waxman S.G., Hoeijmakers J.G., Faber C.G. (2014). Painful neuropathies: The emerging role of sodium channelopathies. J. Peripher. Nerv. Syst..

[289] Lauria G., Ziegler D., Malik R., Merkies I.S., Waxman S.G., Faber C.G., PROPANE Study Group (2014). The role of sodium channels in painful diabetic and idiopathic neuropathy. Curr. Diab. Rep..

[290] Rasakham K., Liu-Chen L.Y. (2011). Sex differences in kappa opioid pharmacology. Life Sci..

[291] Chartoff E.H., Mavrikaki M. (2015). Sex Differences in Kappa Opioid Receptor Function and Their Potential Impact on Addiction. Front. Neurosci..

[292] Smith C.J.W., Ratnaseelan A.M., Veenema A.H. (2018). Robust age, but limited sex, differences in mu-opioid receptors in the rat brain: Relevance for reward and drug-seeking behaviors in juveniles. Brain Struct. Funct..

[293] Dance A. (2019). Why the sexes don’t feel pain the same way. Nature.

[294] Easton A., Norton J., Goodwillie A., Pfaff D.W. (2004). Sex differences in mouse behavior following pyrilamine treatment: Role of histamine 1 receptors in arousal. Pharmacol. Biochem. Behav..

[295] Granados-Soto V., Alonso-López R., Asomoza-Espinosa R., Rufino M.O., Gomes-Lopes L.D., Ferreira S.H. (2001). Participation of COX, IL-1 beta and TNF alpha in formalin-induced inflammatory pain. Proc. West Pharmacol. Soc..

[296] Chichorro J.G., Lorenzetti B.B., Zampronio A.R. (2004). Involvement of bradykinin, cytokines, sympathetic amines and prostaglandins in formalin-induced orofacial nociception in rats. Br. J. Pharmacol..

[297] Dowlati Y., Herrmann N., Swardfager W., Liu H., Sham L., Reim E.K., Lanctôt K.L. (2010). A meta-analysis of cytokines in major depression. Biol. Psychiatry.

[298] Müller N. (2013). The role of anti-inflammatory treatment in psychiatric disorders. Psychiatr. Danub..

[299] Bai Y.M., Chiou W.F., Su T.P., Li C.T., Chen M.H. (2014). Pro-inflammatory cytokine associated with somatic and pain symptoms in depression. J. Affect. Disord..

[300] Post R.M., Uhde T.W., Roy-Byrne P.P., Joffe R.T. (1986). Antidepressant effects of carbamazepine. Am. J. Psychiatry.

